# Adherence to malaria management guidelines by health care workers in the Busoga sub-region, eastern Uganda

**DOI:** 10.1186/s12936-022-04048-2

**Published:** 2022-01-25

**Authors:** Arthur Mpimbaza, Harriet Babikako, Damian Rutazanna, Charles Karamagi, Grace Ndeezi, Anne Katahoire, Jimmy Opigo, Robert W. Snow, Joan N. Kalyango

**Affiliations:** 1 Child Health and Development Centre, Makerere University, College of Health Sciences, Kampala, Uganda; 2 National Malaria Control Division, Ministry of Health, Kampala, Uganda; 3 Department of Paediatrics and Child Health, Makerere University, College of Health Sciences, Kampala, Uganda; 4 Clinical Epidemiology Unit, Makerere University, College of Health Sciences, Kampala, Uganda; 5 Population Health Unit, Kenya Medical Research Institute/Wellcome Trust Research Programme, Nairobi, Kenya; 6 Centre for Tropical Medicine and Global Health, Nuffield Department of Medicine, University of Oxford, Oxford, UK; 7 Department of Pharmacy, Makerere University, College of Health Sciences, Kampala, Uganda

**Keywords:** Malaria case management Uganda, Adherence

## Abstract

**Background:**

Appropriate malaria management is a key malaria control strategy. The objective of this study was to determine health care worker adherence levels to malaria case management guidelines in the Busoga sub-region, Uganda.

**Methods:**

Health facility assessments, health care worker (HCW), and patient exit interview (PEI) surveys were conducted at government and private health facilities in the sub-region. All health centres (HC) IVs, IIIs, and a sample of HC IIs, representative of the tiered structure of outpatient service delivery at the district level were targeted. HCWs at these facilities were eligible for participation in the study. For PEIs, 210 patients of all ages presenting with a history of fever for outpatient care at selected facilities in each district were targeted. Patient outcome measures included testing rates, adherence to treatment, dispensing and counselling services as per national guidelines. The primary outcome was appropriate malaria case management, defined as the proportion of patients tested and only prescribed artemether-lumefantrine (AL) if positive. HCW readiness (e.g., training, supervision) and health facility capacity (e.g. availability of diagnostics and anti-malarials) to provide malaria case management were also assessed. Data were weighted to cater for the disproportionate representation of HC IIs in the study sample.

**Results:**

A total of 3936 patients and 1718 HCW from 392 facilities were considered in the analysis. The median age of patients was 14 years; majority (63.4%) females. Most (70.1%) facilities were HCIIs and 72.7% were owned by the government. Malaria testing services were available at > 85% of facilities. AL was in stock at 300 (76.5%) facilities. Of those with a positive result, nearly all were prescribed an anti-malarial, with AL (95.1%) accounting for most prescriptions. Among those prescribed AL, 81.0% were given AL at the facility, lowest at HC IV (60.0%) and government owned (80.1%) facilities, corresponding to AL stock levels. Overall, 86.9% (95%CI 79.7, 90.7) of all enrolled patients received appropriate malaria case management. However, only 50.7% (21.2, 79.7) of patients seen at PFPs received appropriate malaria management.

**Conclusion:**

Adherence levels to malaria case management guidelines were good, but with gaps noted mainly in the private sector. The supply chain for AL needs to be strengthened. Interventions to improve practise at PFP facilities should be intensified.

**Supplementary Information:**

The online version contains supplementary material available at 10.1186/s12936-022-04048-2.

## Background

Prompt diagnosis and treatment of uncomplicated *Plasmodium falciparum* malaria with artemisinin-based combination therapy (ACT) is a key malaria control and elimination strategy [1]. If not treated promptly, uncomplicated *P. falciparum* malaria has the potential to progress to severe malaria and death [2, 3]. The Ministry of Health (MOH) Uganda adopted (2004) and implemented the ACT treatment policy in 2006, with Artemether-lumefantrine (AL) as the first-line treatment for uncomplicated malaria [4, 5]. However, initially, limited supply and high costs of ACTs impeded translation of policy to clinical practice [5] and adherence levels to the new guidelines remained low. During that period the use of ineffective anti-malarials, such as chloroquine, sulfadoxine-pyrimethamine, and artemisinin monotherapy remained prevalent [6, 7].

In 2010, the World Health Organization (WHO) recommended a policy, shifting from presumptive treatment to mandatory testing of all suspected malaria cases. Prescription of ACT was limited to only those with a positive malaria test and withheld for those with a negative malaria test [8]. In 2011, the Ugandan MOH followed WHO policy [9] to mandate the testing of all fevers as part of malaria case-management guidelines [10] and additionally approved amodiaquine-artesunate (AQ + AS) and dihydroartemisinin-piperaquine (DP) as the alternative and second-line treatments for uncomplicated malaria, respectively [10].

In Uganda, serial demographic health surveys show incremental gains in ACT coverage levels, with the proportion of febrile children receiving an ACT among those who took an anti-malarial increasing to 88% in 2018 from 39% in 2009 [11]. However, data on appropriateness of malaria case management (MCM) among children and adults according to malaria test results remain scarce in Uganda. Previous studies indicate that adherence to the malaria test and treatment policies by health care workers (HCW) were sub-optimal in both public and private facilities [7, 12].

To provide current evidence on malaria case management practice, adherence levels to malaria management guidelines by health care workers at government and private health facilities in the Busoga sub-region; a high transmission setting in eastern Uganda were determined. Facility and HCW factors associated with provision of appropriate malaria case management were also determined.

## Methods

### Design

A cross-sectional design was used to determine levels of adherence to malaria management guidelines and factors associated with provision of appropriate malaria management to outpatients seeking care at government and private health facilities. Using three different survey approaches, data were collected concurrently at each health facility. First, health facility assessments (HFAs) were conducted to determine facility capacity to provide malaria case management service. Second, a health care workers (HCWs) survey was conducted evaluating HCWs competence in providing malaria case management service. Third, patient exit interviews (PEI) centred on malaria management were conducted, detailing type of care provided to patients at the facility.

### Study area

The study was conducted in 11 districts located in Busoga sub-region located in eastern Uganda. The region is reported to have one of the highest malaria parasite prevalence rates in the country, with a population-adjusted posterior mean parasite prevalence of 24.2% (range 9.7–37.6%) [13]. Health service delivery in the sub-region is decentralized at the district level, with outpatient services tiered at three levels; health centre (HC) IIs provide the most basic outpatient services without admissions or a laboratory. For malaria testing, HC IIs rely on RDTs. HC IIIs provide basic outpatient and laboratory service with some inpatient care, while HC IVs additionally provide emergency surgery and blood transfusion service. Based on the 2018 National Health facility inventory [14], the region had 16 HC IVs, 105 HC IIIs, and 325 HCIIs.

## Sample

### Population and eligibility criteria

All HCIVs and IIIs in the Busoga sub-region were eligible for participation, whereas a sample of HCIIs was studied. Government of Uganda (GOU) owned, private not for profit (PNFP), and private for profit (PFP) facilities were eligible for participation. Only facilities that provided general outpatient service and that were functional on the day of the survey were eligible for participation. Sampled, but found to be non-functional HC IIs were replaced by the nearest available HC II of similar ownership status in the same district. Non-functional HC IIIs could not be replaced as all were studied. New HC IIIs not included on the 2018 Health facility inventory automatically qualified. Consenting HCWs involved in providing care to patients at selected facilities were eligible for participation in the HCW survey. HCWs were defined as workers at the facility assigned duties that involved directly or indirectly providing care and services to patients and included the following categories of professionals; doctors, clinical officers, nurses, nursing assistants, and laboratory personnel. The target population for PEIs was febrile patients of all ages exiting outpatient departments after receiving care at an eligible health facility. Eligible patients were those who fulfilled the following criteria: (1) all ages; (2) presenting for an initial outpatient visit at a health facility on survey days; (3) reporting fever during the current illness; (4) not hospitalized or referred to the facility; (5) resident in the district where the facility is located; and (6) who provided consent or assent for children eight years and above.

### Sample size

The study was powered to assess the quality of uncomplicated malaria case management services provided to patients at the district level where authority for managing low level facilities in the district is centred. Therefore, in each district, the sample size was calculated to estimate the proportion of febrile outpatients tested for malaria with a precision of 10%. Assuming that the proportion of febrile patients tested (RDT or microscopy) and treated with AL as per guidelines 75% and considering a design effect of three; a minimum sample size of 210 patients per district was required to estimate the proportion of patients provided appropriate malaria case management with a 95% CI around the estimated frequency of 75%. Therefore, the total sample size was 2310 patients for all the 11 districts. HFA and HCW interviews (self-administered questionnaires for HCWs) were conducted at facilities where PEIs were conducted. At the facility, all HCW involved in management of malaria were eligible for participation.

### Sampling procedures

The MOH Uganda health facility master list (2018), which includes an up-to-date list of all GOU, PNFP, and PFP facilities per district served as the sampling frame for health facilities [14]. The universe of all HCIVs and IIIs were selected for participation. However, due to their abundance in the region [14], 60 out of 325 HCIIs in the sub-region were sampled. The number of HCIIs selected per district was proportional to the number of HCIIs in the district, stratified by ownership status. On the day of the HFA, with the guidance of the facility in-charge a list of eligible HCWs was generated and questionnaires provided to them for completion. PEIs were conducted at each eligible health facility within a district. The number of patients selected per facility was proportional to the facilities outpatient (OP) attendance load in the district, with study participants consecutively enrolled as they exited facilities. As the eligibility criteria included a history of fever for the current illness, all study patients were suspected malaria cases warranting a malaria blood test.

## Indicators

### Malaria case management indicators

The primary indicator of the study was the proportion of all patients provided appropriate malaria case management (MCM) as per Uganda national guidelines [10]. This composite indicator was defined by fulfilling all of three malaria management indicators including (1) tested for malaria by either RDT or microscopy at the health facility, (2) AL prescription if the test is positive, and (3) not prescribing anti-malarials of any type if the test is negative. The primary indicator is also presented based on two different treatment options (1) ACT prescription or (2) AL given, in place of AL prescription among positive patients. Secondary outcome indicators of appropriate MCM included (1) the proportion of patients for whom relevant clinical information was documented, (2) the proportion of patients tested for malaria with an RDT or microscopy; defined as the total number of tested patients divided by all patients, (3) the proportion of patients with a positive malaria test result prescribed an anti-malarial of any type, and (4) anti-malarial types prescribed to patients with a positive malaria test. Additionally, indicators related to anti-malarial prescription practices, dispensing and post dispensing services were reported. Caregiver or patient overall satisfaction with services was ranked on a scale of 0 (lowest) to 10 (highest).

### Health facility indicators

Health facility readiness indicators relevant to malaria case management were considered as potential explanatory variables of the primary outcome. These included: availability of AL and other anti-malarials and malaria testing services (RDT and/or microscopy) at the facility on the day of the survey. Indicators, of malaria case management supervision, health care worker training, availability of guidelines relevant to malaria case management, and job aids pertaining to malaria case management were also calculated as the proportion of facilities that had fulfilled each indicator. The percentage of approved staff positions filled at each facility was calculated by dividing the total number of staff at each facility by the total approved staffing norms for that level of facility [15].

### Health care worker indicators

Indicators for the HCW survey included designation at the facility, proportion who had received in-service training in IMCI and malaria case management in the past 5 years, and proportion who had access to relevant management guidelines. Knowledge indicators related to the test and treat policy were also reported as proportions. These indicators included knowledge that (1) all patients with fever should be tested for malaria, (2) anti-malarials are only prescribed to patients with positive malaria test results, (3) AL is the first-line treatment for uncomplicated malaria, (4) AQ + AS is the alternative first line treatment, and (5) DP is the second line treatment for uncomplicated malaria.

### Data collection procedures

Five teams, each comprised of four to five research assistants led by a senior research assistant experienced in conducting health facility surveys were constituted to collect data. The senior research assistants included one clinical officer, three nurses, and three social scientists. The research assistants were all Year IV medical students from Makerere University and with past experience working in the community. Each team was assigned two districts; an extra district was added to one of the teams, ensuring complete coverage of all 11 districts. Team leaders provided oversight for each group and were responsible for introducing the study to the district health officer and facility in-charges and obtaining authorization to proceed with data collection. Once permission was granted, data collection in the district started. The principal investigator (AM) of the study and project officer provided oversight of all teams. The team leader conducted the HFA using a paper based questionnaire (Additional file 1) after which and in consultation with the facility in-charge identified eligible HCWs for participation in the HCW survey. Upon obtaining consent self-administered paper questionnaires (Additional file 2) were distributed to these HCWs for completion and were collected at a later date. Patient exit interviews were done concurrently or after the HFA and were conducted outside the out-patient department either under an available tent or a makeshift structure strategically located to view patients leaving the facility after receiving care. As patients exited the facility, they were approached by study staff for participation and screened for eligibility criteria. Upon provision of consent, eligible patients were interviewed (Additional file 3) and their medical records scrutinized for information pertaining to their illness and what was done at the facility. Information obtained from medical records included malaria tests and other investigations done and results, anti-malarials and other medicines prescribed and given, and brand types if anti-malarials were given. As a proxy of quality of patient assessment, patient records were also checked to determine if HCWs documented the following clinical parameters (1) patients’ age, (2) history of prior use of anti-malarials during the current illness episode, (3) temperature, and (4) weight. For children aged 6 months or less, caregivers were asked and children evaluated for danger signs. Among patients given AL at the facility, data on counselling services including how and when to take medicines, and what to do if the child vomited medicines or experienced an adverse reaction were also documented based on the respondents report. At the end of the interview, caregivers and older patients were asked to rank their overall level of satisfaction with provided services on a scale of 0 (lowest) to 10 (highest). PEI data were collected electronically using the Open Data Kit (ODK) software installed on Samsung Galaxy tablets. Each phone was enabled to capture GPS coordinates to provide facility locations. ODK data were uploaded to a central server based at the College of Health Sciences, Makerere University, where they were checked for completeness by a data manager.

### Data analysis

Data were analysed using STATA (version 14; STATA Corp., College Station, TX, USA). Survey data were weighted to ensure that the study sample was representative of the population. As the number of children sampled per facility was proportionate to the facilities patient load, the weighting strategy only focused on ensuring that representation of facilities in the study sample matched the distribution of facilities in the region. Data collected from DHIS-2 was used to determine the probability of sampling HC IV, III, and IIs. The inverse of the probability of a facility being sampled defined the weight for each level of facility (Additional file 4). Patient, health facility, and HCW characteristics were summarized as proportions or medians for categorical and continuous data, respectively. Indicators of malaria case management were summarized as percentages stratified by facility type and age group. Difference between indicators across facility types and age group were reported as the absolute percentage difference with corresponding 95% CIs included, and adjusted for clustering at the health facility level. The significance (p < 0.05) of differences was tested using the chi square test. The Generalized estimating equation (GEE) model was used to identify independent patient, facility, and health care worker factors associated with appropriate malaria case management at each facility. Covariates with a significant (p < 0.150) association with the outcome at univariate analysis were included in the final model. Robust standard errors specifying clustering at the health facility-level were used to compute 95% confidence intervals. Using the binomial distributional family and logit link function the coefficients and corresponding standard errors and confidence intervals were exponentiated as odds ratios. Using Stata’s QIC program [16], the quasilikelihood under the independence model criterion (QIC) was used to select the model with the best correlation structure (QIC = 1481 and QIC_u = 1422).

## Results

### Selection of patients, health care workers, and health facilities

**Fig. 1:**
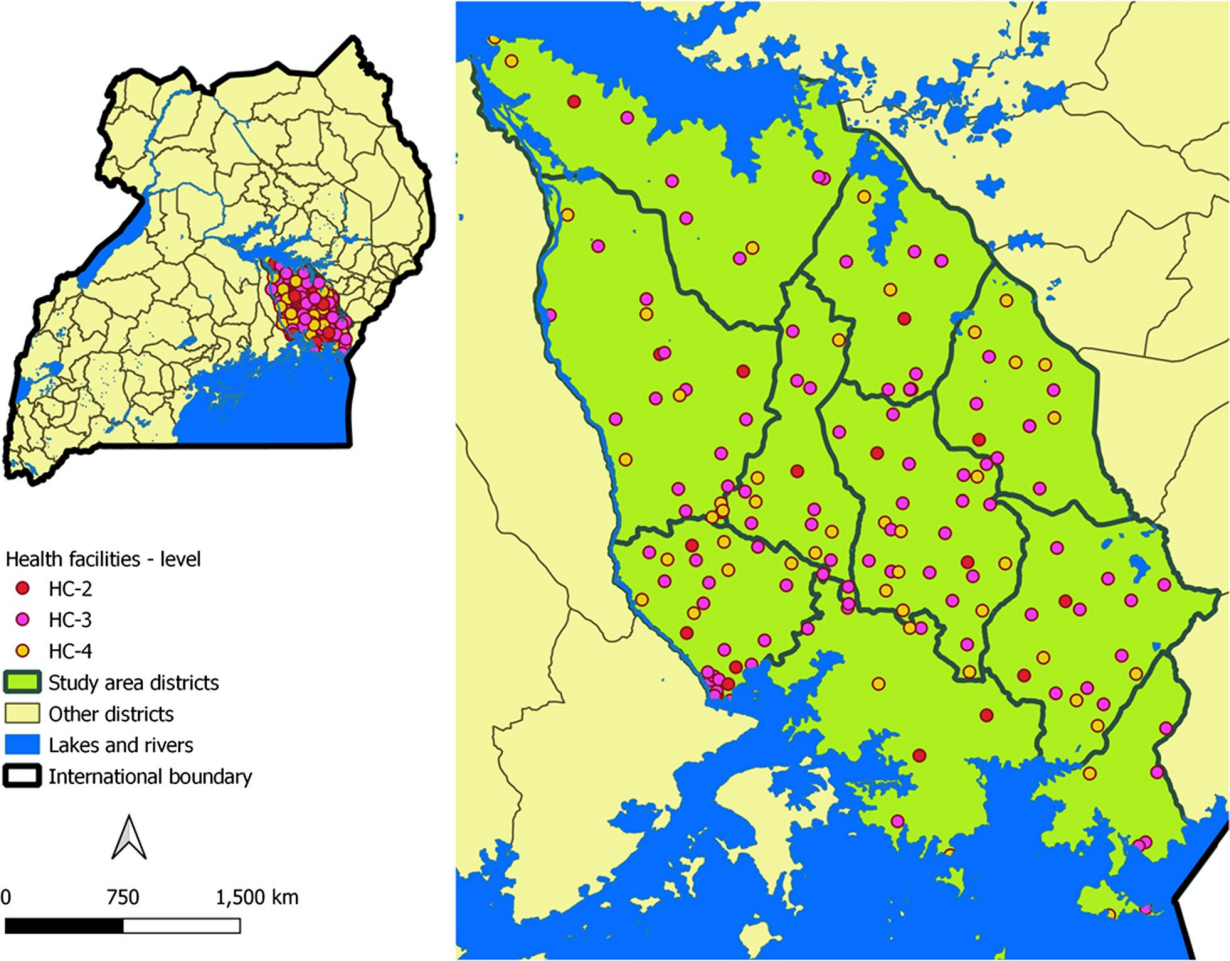
Study site

For PEIs, 2350 patients were screened, of whom 40 were excluded, leaving 2310 patients eligible for participation. Presenting with an illness not associated with fever (25; 62.5%) was the most frequent reason for exclusion (Additional file 5). Only facilities with complete data, representing the facility, HCWs, and patients (PEIs) were considered in the final analysis. Consequently, the final eligible study sample comprised of 172 health facilities (Fig. 1), where all aspects of the study; HFA (172 facilities), PEI (2196 patients) and HCW interviews (1086) were conducted (Additional file 6). Details of how sites were sampled, enrolled, excluded, and replaced are provided in Additional file 7). The number of questionnaires not returned by HCW eligible for the survey was not tracked. However, using onsite (on the survey day) staffing level data determined during the HFA, HCW response rates among targeted HCWs ranged between 30 to 81%, mean 70% (Additional file 8).

### Baseline characteristics: patients, health facilities, and health care workers

Majority (63.4%) of patients were females, a pattern consistent across facility types (Table 1). The median age of all patients was 14 (IQR 3, 27) years, with older patients seen at PFP (Median 23, IQR 3, 28) facilities. Most (70.1%) facilities were HCIIs and owned by GOU (72.7%). Facility supervisory visits that included malaria case management practice conducted during the current or preceding year of the survey were uncommon (Table 1). Overall, malaria testing (microscopy or RDT) services were available at most (90.6%) facilities. On the contrary, availability of AL tablet packs of any type (76.5%) was less common (Table 1), especially at HC IVs (75.0%) and IIs (72.7%). However, considering ownership status, PNFP (93.4%) facilities were more likely to have AL tablet packs of any type than GOU (71.6%) and PFP (80.7%) facilities. Overall, and with the exception of Uganda Clinical Guidelines (UCG) which was relatively common, other guidelines including the Integrated Management of Malaria (IMM) and Integrated Management of Childhood Illnesses (IMCI) guidelines were uncommon. Overall, the median percentage of staff vacancies that were filled was 56% (IQR 33%, 78%), higher at HC III and GOU facilities (Table 1). Of 1718 interviewed HCWs, majority (48.3%) were enrolled nurses followed by nursing aids (22.0%). Most (> 90.0%) HCWs were knowledgeable of malaria treatment guidelines including 1) testing all febrile patients; 2) prescribing anti-malarials to only patients with a positive test result; and 3) AL is the recommended first-line treatment for uncomplicated malaria (Table 1).

**Table 1:** Characteristics of patients, facilities, and providers in the Busoga sub-region, 2020

Variable	All	Level of heath facility			Owner of health facility		
		IV	III	II	GOU	PNFP	PFP
Patient characteristics							
Number of patients	3936	363	1398	2175	3414	323	199
Females, n (%)	2497 (63.4)	229 (63.0)	873 (62.4)	1395 (64.1)	2193 (64.2)	193 (59.7)	111 (55.8)
Age in years, median (IQR)	14 (3, 27)	10 (2, 25)	14 (3, 27)	14 (3, 27)	13 (3, 26)	14 (2, 25)	23 (3, 28)
Age category							
< 5 years, n (%)	1235 (31.4)	126 (34.7)	429 (30.7)	680 (31.3)	1057 (31.0)	116 (35.9)	62 (31.2)
5 to < 15 years, n (%)	776 (19.7)	74 (20.3)	287 (20.5)	415 (19.1)	714 (20.9)	46 (14.2)	16 (8.0)
> 15 to 45 years, n (%)	1643 (41.7)	141 (38.8)	552 (39.5)	950 (43.7)	1394 (40.8)	144 (44.6)	105 (52.8)
> 45 years, n (%)	282 (7.2)	22 (6.1)	130 (9.3)	130 (5.9)	249 (7.3)	17 (5.3)	16 (8.0)
High (> 37.5 °C) temperature, n (%)	1267 (32.2)	144 (39.7)	373 (26.7)	750 (34.5)	1106 (32.4)	103 (31.9)	58 (29.2)
Illness duration (days), median (IQR)	3 (2, 5)	3 (3, 6)	3 (2, 5)	3 (2, 4)	3 (2, 5)	3 (2, 6)	3 (2, 3)
Prior use of anti-malarials, n (%)	631 (16.0)	59 (16.2)	242 (17.3)	330 (15.1)	537 (15.7)	47 (14.5)	47 (23.6)
Health facility characteristics							
Number of facilities	392	16	101	275	285	76	31
Supervised in MCM 2019/2020, n (%)	59 (15.1)	4 (25.0)	25 (24.7)	30 (10.9)	35 (12.3)	16 (21.1)	8 (25.8)
Availability of equipment/medicines							
Weighing scale, n (%)	340 (86.7)	16 (100.0)	99 (98.0)	225 (81.8)	238 (83.5)	71 (93.4)	31 (100.0)
Thermometer, n (%)	255 (65.0)	12 (75.0)	93 (92.0)	150 (54.5)	158 (55.4)	66 (86.8)	31 (100.0)
Malaria test, n (%)	355 (90.6)	16 (100.0)	99 (98.0)	240 (87.2)	253 (88.8)	71 (93.4)	31 (100.0)
AL pack of any type in stock, n (%)	300 (76.5)	12 (75.0)	88 (87.1)	200 (72.7)	204 (71.6)	71 (93.4)	25 (80.7)
Availability of guidelines/charts							
IMM guidelines 2014, n (%)	92 (23.5)	4 (25.0)	38 (37.6)	50 (18.2)	65 (22.8)	19 (25.0)	8 (25.8)
IMCI guidelines, n (%)	136 (34.7)	9 (56.3)	67 (66.3)	60 (21.8)	109 (38.3)	24 (31.6)	3 (9.7)
UCG, n (%)	276 (70.4)	15 (93.8)	91 (90.1)	170 (61.8)	`212 (74.4)	55 (72.4)	9 (29.0)
AL dosing procedures, n (%)	98 (25.0)	8 (50.0)	45 (44.5)	45 (16.4)	78 (27.4)	14 (18.4)	6 (19.4)
% of vacancies filled, median (IQR)	56 (33, 78)	76 (70, 85)	79 (63, 95)	56 (33, 67)	66 (44, 79)	50 (33, 67)	55 (33, 67)
Health care worker characteristics							
Number of health care workers, N	1718	219	709	790	1349	251	118
Position							
Doctor, n (%)	11 (0.6)	7 (3.2)	4 (0.6)	0	8 (0.6)	2 (0.8)	1 (0.9)
Clinical Officer, n (%)	174 (10.1)	39 (17.8)	110 (15.5)	25 (3.2)	145 (10.8)	21 (8.4)	8 (6.8)
Registered nurse, n (%)	241 (14.0)	47 (21.5)	114 (16.1)	80 (10.1)	205 (15.2)	33 (13.2)	3 (2.5)
Enrolled nurse, n (%)	831 (48.3)	94 (42.9)	332 (46.8)	405 (51.3)	622 (46.1)	125 (49.8)	84 (71.2)
Nursing assistant, n (%)	378 (22.0)	21 (9.6)	107 (15.1)	250 (31.7)	303 (22.5)	56 (22.3)	19 (16.1)
Laboratory staff, n (%)	36 (2.1)	5 (2.3)	26 (3.7)	5 (0.6)	28 (2.1)	6 (2.4)	2 (1.7)
Other, n (%)	47 (2.7)	6 (2.7)	16 (2.3)	25 (3.1)	38 (2.8)	8 (3.2)	1 (0.9)
In service training in the past 5 years							
IMCI, n (%)	583 (36.7)	70 (32.0)	283 (39.9)	230 (29.1)	447 (33.1)	90 (35.9)	46 (39.0)
MCM, n (%)	628 (36.6)	87 (39.7)	296 (41.7)	245 (31.0)	486 (36.0)	93 (37.0)	49 (41.5)
Access to guidelines							
IMM guidelines, n (%)	1064 (61.9)	134 (61.2)	500 (70.5)	430 (54.4)	879 (65.2)	141 (56.2)	44 (37.3)
UCG, n (%)	1442 (83.9)	173 (79.0)	634 (89.4)	635 (80.4)	1189 (88.1)	181 (72.1)	72 (61.0)
Knowledge of malaria treatment policy							
Malaria test for all fevers, n (%)	1634 (95.1)	210 (95.9)	669 (94.4)	755 (95.6)	1279 (94.8)	239 (95.2)	116 (98.3)
Anti-malarials to only positives, n (%)	1624 (94.5)	213 (97.2)	666 (93.9)	745 (94.3)	1281 (94.9)	230 (91.6)	113 (95.7)
1st line treatment for UM, n (%)	1555 (90.5)	200 (91.3)	655 (92.4)	700 (88.6)	1237 (91.7)	227 (90.4)	91 (77.1)
Alt. 1st line treatment for UM, n (%)	332 (19.3)	23 (10.5)	164 (23.1)	145 (18.4)	246 (18.2)	84 (33.5)	2 (1.7)
2nd line Rx for UM, n (%)	463 (27.0)	63 (28.8)	265 (37.4)	135 (17.1)	380 (28.2)	73 (29.1)	10 (8.5)

*MCM* malaria case management, *IMM* integrated malaria management, *IMCI* integrated management of childhood illness, *UCG* Uganda clinical guidelines, *Alt* alternative, *UM* uncomplicated malaria

### Uncomplicated malaria case management indicators

Majority (91.8%) of patients had age documented on their medical record. However, history of prior use of anti-malarials during the current illness (5.4%) was rarely documented (Table 2). At facilities where thermometers and weighing scales were available, temperature and weight were documented in 24.3% and 79.7% of all patient records, respectively (Table 2). Overall, malaria testing rates were high (90.2%), with insignificant variation across facility levels. However, compared to patients seen at GOU (91.6%) and PNFP (89.8%) facilities, those seen at PFP (67.3%) facilities were less likely to be tested, albeit not significantly. The overall test positivity rate (TPR) was 59.3%, highest at HCII (63.0%) and GOU (60.3%) facilities. Compared to all other age groups, TPRs were significantly (p < 0.001) higher (75.6%) among patients aged 5 to 14 years (Table 2). Anti-malarials were rarely (1.7%) prescribed to patients with negative test results. However, prescription rates of anti-malarials to patients not tested was high especially at HC IIs, PNFP, and PFP facilities (Table 3). Overall, AL (95.1%) was the most frequently prescribed anti-malarial to patients with a positive malaria test result, a practise that was consistent across all facility levels. However, compared to GOU (96.6%; difference 41.0%, 95% CI 11.6, 70.3; p = 0.006) and PNFP (95.6%; difference 40.0%, 95% CI 10.4, 69.6; p = 0.008) facilities, prescription of AL to patients with a positive test result was less common at PFP (55.6%) facilities. Intravenous artesunate (20.8%) and dihydroartemisinin-piperaquine (20.8%) accounted for most of other types prescribed at PFP facilities.

**Table 2:** Uncomplicated malaria case management indicators among patients exiting facilities in the Busoga sub-region

Variable	All	Level			Ownership			Age category			
		IV	III	II	GOU	PNFP	PFP	0–4	5 -14	14–44	> 45
Total number of patients enrolled, N	3936	363	1398	2175	3414	323	199	1235	776	1643	282
Documentation of information											
Age, n (%)	3612 (91.8)	324 (89.3)	1298 (92.9)	1990 (91.5)	3131 (91.7)	292 (90.4)	189 (94.9)	1156 (93.6)	715 (92.1)	1476 (89.8)	265 (94.0)
Prior use of anti-malarials, n (%)	214 (5.4)	5 (1.4)	69 (4.9)	140 (6.4)	136 (3.9)	42 (13.0)	36 (18.1)	45 (3.6)	39 (5.0)	109 (6.6)	21 (7.4)
Temperature, n (%)	649 (24.3)	34 (11.6)	385 (29.9)	230 (21.0)	440 (20.2)	127 (43.3)	82 (41.2)	221 (25.9)	114 (22.0)	274 (24.1)	40 (24.2)
Weight, n (%)	2842 (79.7)	245 (67.5)	1147 (83.7)	1450 (79.0)	2490 (81.1)	249 (83.5)	103 (51.8)	891 (80.6)	586 (81.9)	1150 (77.8)	215 (79.9)
Malaria testing											
Suspects tested, n (%)	3552 (90.2)	317 (87.3)	1330 (95.1)	1905 (87.6)	3128 (91.6)	290 (89.8)	134 (67.3)	1121 (90.8)	690 (88.9)	1488 (90.6)	253 (89.7)
Test positivity rate, n (%)	2106 (59.3)	191 (60.3)	715 (53.8)	1200 (63.0)	1894 (60.5)	139 (47.9)	73 (54.5)	715 (63.8)	522 (75.6)	749 (50.3)	120 (47.4)
Malaria test: RDT, n (%)	3040 (85.6)	124 (39.1)	1086 (81.6)	1830 (96.1)	2721 (87.0)	246 (84.8)	73 (54.5)	959 (85.6)	600 (87.0)	1265 (85.0)	216 (85.4)
Anti-malarial treatment prescribed											
Among tested positive, n (%)	2094 (99.4)	190 (99.5)	704 (98.5)	1200 (100.0)	1886 (99.6)	136 (97.8)	72 (98.6)	713 (99.7)	520 (99.6)	744 (99.3)	117 (97.5)
Among tested negative, n (%)	25 (1.7)	1 (0.8)	9 (1.5)	15 (2.1)	22 (1.8)	3 (2.0)	0	19 (4.7)	2 (1.2)	3 (0.4)	1 (0.8)
Among not tested, n (%)	216 (56.3)	10 (21.7)	16 (23.5)	190 (70.4)	134 (46.9)	22 (66.7)	60 (92.3)	80 (70.2)	68 (79.1)	56 (36.1)	12 (41.4)
Anti-malarial types prescribed											
AL	1991 (95.1)	175 (92.1)	671 (95.3)	1145 (95.4)	1821 (96.6)	130 (95.6)	40 (55.6)	667 (93.6)	498 (95.7)	710 (95.4)	116 (99.1)
AL + SP	1 (0.05)	1 (0.5)	0	0	1 (0.1)	0	0	1 (0.1)	0	0	0
AL + TQ	1 (0.05)	0	1 (0.1)	0	1 (0.1)	0	0	1 (0.1)	0	0	0
AL + MQ	5 (0.2)	0	0	5 (0.4)	5 (0.3)	0	0	0	5 (0.9)	0	0
IV AS + AL	30 (1.4)	6 (3.0)	14 (2.0)	10 (0.8)	29 (1.5)	1 (0.7)	0	16 (2.2)	12 (2.3)	1 (0.1)	1 (0.9)
DP	33 (1.6)	4 (2.1)	9 (1.3)	20 (1.7)	17 (0.9)	1 (0.7)	15 (20.8)	9 (1.3)	3 (0.6)	21 (2.8)	0
IM rtemether	2 (0.1)	0	2 (0.3)	0	0	1 (0.7)	1 (1.4)	1 (0.1)	1 (0.2)	0	0
Piperaquine	5 (0.2)	0	0	5 (0.4)	5 (0.3)	0	0	0	0	5 (0.7)	0
Quinine	1 (0.05)	0	1 (0.1)	0	0	0	1 (1.4)	1 (0.1)	0	0	0
Amodiaqu ne	1 (0.05)	0	1 (0.1)	0	0	1 (1.5)	0	0	0	1 (0.1)	0
Oral rtesunate	1 (0.05)	1 (0.5)	0	0	1 (0.1)	0	0	1 (0.1)	0	0	0
IV AS	23 (1.1)	3 (1.6)	5 (0.7)	15 (1.2)	6 (0.5)	2 (3.1)	15 (20.8)	16 (2.2)	1 (0.2)	6 (0.8)	0
Appropriate MCM; AL prescribed, n (%)	3419 (86.9)	301 (82.9)	1278 (91.4)	1820 (84.6)	3040 (89.1)	278 (86.1)	101 (50.7)	1056 (85.5)	669 (86.2)	1446 (88.0)	248 (87.9)
Appropriate MCM; ACT prescribed, n (%)	3464 (88.0)	306 (84.3)	1298 (92.9)	1860 (85.5)	3065 (89.8)	282 (87.3)	117 (58.8)	1067 (86.4)	674 (86.8)	1472 (89.6)	251 (89.0)
Appropriate MCM; AL given, n (%)	3034 (77.1)	230 (63.4)	1134 (81.1)	1670 (76.8)	2671 (78.2)	268 (82.9)	95 (47.7)	931 (75.4)	566 (72.9)	1320 (80.3)	217(77.0)
Patient satisfaction score^1^, median (IQR)	8 (6, 9)	7 (6, 8)	8 (6, 9)	8 (7, 9)	8 (6, 9)	8 (7, 9)	9 (8, 10)	8 (7, 9)	8 (6, 9)	8 (6, 9)	8 (6, 9)

*AL* Artemether-Lumefantrine, *ACT* Artemisinin-based Combination Therapy, *MCM* malaria case management, *SP *Sulfadoxine Pyrimethamine, *TQ *Tafenoquine, *MQ *Mefloquine, *IV* *AS *intravenous artesunate, *DP *Dihydroartemisinin Piperaquine

^1^Score; range 0 (least) to 10 (most);

**Table 3:** Factors associated with appropriate malaria case management

Variable	Number of patients appropriately managed for malaria N = 3509	Univariate analysis		Multivariate analysis	
		OR (95% CI)	p-value	OR (95% CI)	p-value
Patient characteristics					
Females, n (%)	2200 (62.7)	0.95 (0.84, 1.07)	0.409		
Age in years	NA	1.01 (1.01, 1.02)	< 0.001	1.01 (1.01, 1.02)	0.002
Prior use of anti-malarials, n (%)	568 (16.2)	0.85 (0.73, 0.99)	0.05		
Documentation of information on patient record					
Age and medicine use, n (%)	3220 (91.7)	1.70 (1.14, 2.51)	0.008	0.87 (0.65, 1.15)	0.342
Temperature and weight, n (%)	2744 (78.2)	1.98 (1.55, 2.54)	< 0.001	2.07 (1.46, 2.93)	< 0.001
Patient satisfaction score	NA	1.06 (1.03, 1.10)	< 0.001	1.12 (1.06, 1.18)	< 0.001
Health facility characteristics					
Level					
Health centre IV	303 (8.6)	1		1	
Health centre III	1316 (37.5)	1.47 (0.68, 3.30)	0.340	6.86 (1.57, 29.8)	0.010
Health centre II	1890 (53.9)	0.87 (0.40, 1.88)	0.735	8.29 (2.36, 29.0)	0.001
Health centre III#AL	NA			0.15 (0.02, 0.95)	0.045
Health centre II#AL	NA			0.14 (0.28, 0.71)	0.018
Owner					
GOU	3080 (87.7)	1		1	
PNFP	322 (9.2)	0.73 (0.41, 1.32)	0.308	0.55 (0.32, 0.97)	0.041
PFP	107 (3.0)	0.25 (0.13, 0.49)	< 0.001	0.21 (0.10, 0.42)	< 0.001
Supervised in MCM 2019/2020	641 (18.3)	4.80 (2.36, 9.75)	< 0.001	4.39 (1.62, 11.9)	0.003
Availability of equipment / medicines					
Weighing scale, n (%)	3226 (92.8)	1.92 (0.99, 3.73)	0.051		
Thermometer, n (%)	2420 (68.9)	1.37 (0.86, 2.18)	0.179		
AL in stock on survey day, n (%)	2591 (73.8)	0.51 (0.27, 0.94)	0.032	2.27 (0.52, 9.85)	0.271
Malaria test on survey day, n (%)	3332 (94.9)	2.49 (1.23, 5.00)	0.011	7.73 (3.84, 15.6)	< 0.001
Availability of guidelines/charts					
IMM 2014, n (%)	933 (26.6)	0.89 (0.52, 1.53)	0.686		
IMCI guidelines, n (%)	1762 (0.50)	2.49 (1.55, 3.99)	< 0.001	0.88 (0.51, 1.52)	0.670
UCG guidelines, n (%)	2908 (82.9)	4.39 (2.76, 6.98)	< 0.001	3.28 (1.96, 5.49)	< 0.001
AL dosing procedures, n (%)	1183 (33.7)	1.58 (0.84, 2.96)	0.153		
% of vacancies filled	NA	1.36 (0.44, 4.19)	0.587		
Health care worker characteristics					
Position					
Doctor, n (%)	9 (0.3)	1			
Clinical Officer, n (%)	286 (8.2)	1.17 (0.38, 3.62)	0.777		
Nurse-degree, n (%)	24 (0.7)	3.24 (0.58, 17.9)	0.177		
Nurse diploma, n (%)	2094 (59.7)	1.38 (0.43, 4.37)	0.580		
Nursing Assistant, n (%)	975 (27.8)	1.39 (0.44, 4.41)	0.571		
Lab personnel	55 (1.6)	1.66 (0.50, 5.48)	0.403		
Other, n (%)	66 (1.9)	1.52 (0.45, 5.13)	0.499		
Supervised in MCM in the past 3 months, n (%)	1307 (37.3)	1.46 (1.20, 1.77)	< 0.001	1.64 (1.18, 2.28)	0.003
In service training in the past 5 years					
IMCI, n (%)	1379 (39.3)	0.84 (0.70, 0.99)	0.047		
MCM, n (%)	1245 (35.7)	0.80 (0.66, 0.96)	0.021		
Access to guidelines					
IMM guidelines, n (%)	2023 (57.6)	1.10 (0.91, 1.32)	0.311		
UCG, n (%)	3067 (87.4)	1.59 (1.24, 2.05)	< 0.001	1.25 (0.90, 1.72)	0.174
Knowledge of malaria treatment policy					
Malaria test for all fevers, n (%)	3375 (96.2)	0.84 (0.57, 1.24)	0.400		
Anti-malarials to only positives, n (%)	3374 (96.4)	1.41 (0.88, 2.25)	0.153		
1st line treatment for UM, n (%)	3168 (90.3)	0.84 (0.63, 1.11)	0.230		

*MCM* malaria case management, *IMM* Integrated Malaria Management, *IMCI* Integrated Management of Childhood Illness, *UCG* Uganda Clinical Guidelines, *UM *Uncomplicated malaria

Of all enrolled patients, 86.9% (95% CI 80.5, 91.3) were appropriately (considering AL prescription as the treatment of choice) managed for malaria. Compared to patients seen at GOU (89.1%; difference 38.4%, 95% CI 4.4, 72.1; p = 0.027) and PNFP (86.1%; difference 35.4%, 95% CI − 0.34, 70.9; p = 0.052) facilities, patients seen at PFP (50.7%) facilities were less likely to receive appropriate malaria case management (Table 2). When appropriate malaria management was defined based on prescription of ACT and not just AL, differences in performance between GOU, PNFP, and PFP facilities were large but not significant. However, when appropriate management was defined based on AL received and not just prescribed, patients seen at PNFP (82.9%) facilities were significantly more likely to receive appropriate care compared to those seen at PFP (47.7%; difference 35.2%, 95% CI 1.3, 69.2; p = 0.042) facilities. Overall, most respondents ranked their satisfaction with provided services highly (Table 2).

### Factors associated with appropriate malaria case management

Based on univariate analysis, increase in age was associated with higher likelihood of getting appropriate malaria management (Table 3). Documentation of (1) age and history of anti-malarial use prior to presentation at the facility and (2) temperature and weight on the patient’s medical record form were associated with appropriate management. Facilities that had (1) a malaria case management supervisory visit (OR 4.80, 95% CI 2.36, 9.75; p < 0.001), (2) malaria testing services (OR 2.49, 95% CI 1.23, 5.00; p = 0.011), (3) IMCI (OR 2.49, 95% CI 1.55, 3.99; p < 0.001) guidelines, and (4) Uganda Clinical Guidelines (UCG; OR 4.39, 95% CI 2.76, 6.98; p < 0.001) were associated with patients receiving appropriate malaria case management. Increase in satisfaction levels with provided services was associated with receiving appropriate care. However, staffing level did not have a significant association with appropriate malaria case management. HCW who had received training in IMCI or MCM was associated with lower likelihood of appropriate malaria case management (Table 3).

Upon adjusting for confounders and inclusion of a significant (p = 0.05) interaction term between health facility level and availability of AL at the facility in the final model (Table 3), increasing patient age and documentation of patient temperature and weight maintained association with appropriate malaria case management. Additionally, facilities that had (1) a malaria case management supervisory visit, (2) malaria testing services, (3) IMCI guidelines, and (4) UCG maintained their association with appropriate malaria case management (Table 3). Compared to HC IVs, HC III (OR 6.86, 95% CI 1.57, 29.8; p = 0.010) and IIs (OR 8.29, 95% CI 2.36, 29.0; p = 0.001) were more likely to provide appropriate care. On the contrary, compared to GOU facilities, PFP (OR 0.21, 95% CI 0.10, 0.42; p < 0.001) and PNFP (OR 0.55, 95% CI 0.32, 0.97; p = 0.041) facilities were less likely to provide appropriate care (Table 3). HCWs supervised on malaria case management were also associated with appropriate malaria case management (Table 3). A significant (p = 0.048) interaction term was noted between HCWs trained in IMCI and MCM. Upon inclusion of the interaction term in the model, the association between both trainings and the outcome became insignificant. Both variables were excluded from the final model as they did not add any value to the model. Results did not change much when the outcome, appropriate malaria case management, was defined based on different treatment options; prescription of an ACT or AL given among patients with a positive test (Additional file 9).

### Quality of AL prescribing, dispensing, and counseling practices

Among 1991 patients with a positive malaria test result and prescribed AL, 1613 (81.0%) received AL at the facility (Table 4). Patients seen at HCII (85.6%) facilities were more likely to have received AL as compared to those seen at HCIVs (60.0%; difference 25.5%) and HCIIIs (78.6%; difference 6.9%) facilities but differences were not statistically significant. Patients prescribed AL at PNFP (92.3%) facilities were more likely to have received AL at the facility compared to those seen at GOU (80.1%; difference 12.1%, 95%CI − 0.68, 29.3, p = 0.063) facilities, albeit non-significantly (Table 4). Among the 1613 patients who given AL, treatment administration at the facility was rare (3.2%), even among young children under five years of age (4.6%). In contrast, patients and caregivers of children were frequently counseled on how to take medicines while at home (88.3%), to take medicines after a meal (75.5%), and to complete treatment (71.8%). Response to drug adverse events or vomiting soon after taking medicines was rarely discussed with patients. Considering patients given dispersible AL, very few caregivers were given water to mix the medicine or given instruction on how to prepare the medicine. Patterns were consistent across all facility types (Table 4).

**Table 4:** Prescription, administration, and counselling service of AL among patients with a positive malaria test result

Variable	All	Level			Ownership			Age category			
		IV	III	II	GOU	PNFP	PFP	0–4	5–14	14–44	> 45
AL prescribed	1991	175	671	1145	1821	130	40	667	498	710	116
AL given, n (%)	1613 (81.0)	105 (60.0)	528 (78.6)	980 (85.6)	1459 (80.1)	120 (92.3)	34 (85.0)	544 (81.5)	400 (80.3)	584 (82.3)	85 (73.3)
Of those given AL											
Given single blister pack AL, n (%)	1147 (71.1)	76 (72.3)	351(66.4)	720 (73.5)	1035 (70.9)	85 (70.8)	2711 (79.4)	420 (77.2)	90 (22.5)	554 (94.9)	83 (97.6)
1st dose taken at facility, n (%)	52 (3.2)	2 (1.9)	15 (2.6)	35 (3.6)	37 (2.5)	10 (8.3)	5 (14.7)	25 (4.6)	12 (3.0)	15 (2.6)	0
1st dose swallowed in front of HW, n (%)	14 (26.9)	0	4 (0.7)	10 (28.6)	9 (24.3)	5 (50.0)	0	8 (32.0)	1 (8.3)	5 (33.3)	0
Counseling to those given AL											
How to take medicine at home, n (%)	1432 (88.3)	97 (92.3)	460 (87.1)	875 (89.3)	1303 (89.3)	100 (83.3)	29 (85.3)	477 (87.7)	346 (86.5)	531 (90.9)	78 (91.8)
Next dose after 8 h, n (%)	875 (54.2)	44 (41.9)	261 (49.4)	570 (58.1)	796 (54.6)	50 (41.7)	29 (85.3)	293 (53.9)	212 (53.0)	309 (52.9)	61 (71.7)
Take medicine after meals, n (%)	1250 (77.5)	68 (64.7)	402 (76.1)	780 (79.6)	1120 (76.7)	96 (80.0)	34 (100.0)	407 (74.8)	304 (76.0)	468 (80.1)	71 (83.5)
Complete treatment, n (%)	1159 (71.8)	72 (68.6)	377 (71.4)	710 (72.5)	1044 (71.6)	82 (68.3)	33 (97.1)	396 (72.8)	278 (69.5)	426 (72.9)	59 (69.4)
Response to drug AE, n (%)	83 (5.2)	3 (2.9)	30 (5.7)	50 (5.1)	76 (5.2)	7 (5.8)	0	33 (6.1)	19 (4.8)	28 (4.8)	3 (3.5)
Response after vomiting, n (%)	2 (0.1)	0	2 (0.4)	0	2 (0.1)	0	0	2 (0.4)	0	0	0
Given dispersible AL	267 (24.3)	17 (22.3)	90 (25.6)	160 (22.2)	249 (24.1)	18 (21.2)	0	251 (59.7)	15 (16.7)	1 (0.2)	0
If yes, given water, n (%)	16 (6.0)	0	6 (6.7)	10 (6.2)	15 (6.0)	1 (5.6)	0	15 (6.0)	0	1 (100.0)	0
If yes, Mother given instructions, n (%)	10 (3.8)	0	5 (5.6)	3 (3.1)	9 (3.6)	1 (5.6)	0	9 (3.6)	0	1 (100.0)	0

## Discussion

High adherence levels to the test and treat components of malaria case management guidelines by HCW in the Busoga sub-region were noted in this study. Most patients were tested (90.2%) for malaria and of those with a positive result, AL (95.1%; Table 2) was the most frequently prescribed anti-malarial. Intravenous artesunate and DP accounted for most of other antimalarial types prescribed. Very few patients with negative results had anti-malarials prescribed (1.8%). Compared to previous studies in the same region [7, 17], these results reflect a significant improvement in HCW adherence to malaria case management guidelines; consistent with reports from other countries in SSA documenting improved case-management practices over the last decade [18, 19]. However, amidst progress, gaps were noted. At HCIV (87.3%), HCII (87.5%), and at PFP (87.5%) facilities, testing rates were below the set target of 100% as indicated in the 2014–2020 Uganda malaria reduction strategic plan [20] (Table 2). Additionally, at PFPs, prescription of anti-malarials other than AL was common. Health facilities that had a supervisory visit involving malaria case management and those that had Uganda Clinical Guidelines (UCG) were more likely to provide appropriate malaria case management.

Testing rates were generally found to be high in this study, with RDTs being the most frequent test type (85.6%; Table 2). However, testing rates were below the set target of 100% at HCIV and HC II facilities. All HC IVs had malaria tests and were adequately staffed as has been recently reported in a survey of facilities in Uganda [21], excluding inadequate staffing as a possible reason for not testing. Similarly, only two of nine facilities where patients were not tested did not have RDTs on the day of the assessment. As testing services were often available at most facilities including those where patients were not tested, reasons other than lack of RDTs or microscopy services, such as large patient volumes, could explain lack of testing at some facilities. Malaria testing rates were low at PFP (67.3%) facilities, a recognized problem of PFP facilities [12, 22–25]. Ironically, all PFP had malaria test services being offered on the day of the survey, contrasting with previous studies indicating limited availability of malaria testing services at PFP facilities [12]. Low testing rates at PFP could be explained by patient concerns about cost and time spent testing [23, 26].

AL was the most frequently prescribed anti-malarial to patients with positive results. However, use of DP and intravenous artesunate, recommend for severe malaria was also common, especially at PFPs. Prescription of non-recommended anti-malarials to outpatients at PFP facilities has been described before and has been attributed to lack of knowledge, profit driven practice, or sometimes patient demand for specific treatments [27]. Use of parenteral anti-malarials among outpatients is unacceptable, increasing the likelihood of under dosing patients, subsequently treatment failure and poor outcomes, and selection of artemisinin resistant parasites [28]. The unjustified use of IV artesunate in the private sector has previously been described in western Uganda [29], calling for urgency in monitoring of irrational use of parenteral anti-malarials, particularly in the private sector. Prescription of non-artemisinin monotherapies was very rare (< 2%; Table 2), just like was the prescription of anti-malarials to patients with negative test results. A negligible proportion (0.6%) of patients with positive malaria test results were not prescribed an anti-malarial. This peculiar finding has been described in a review of eight studies from five different countries in SSA, where 7% of patients with a positive RDT were not prescribed an ACT or anti-malarial [30]. Studying reasons why patients with a positive test result were not prescribed an anti-malarial was beyond the scope of the study. However, among patients testing positive for malaria but not prescribed an anti-malarial most tests done were RDTs. A recognized limitation of RDTs is continued detection of antigens (giving false positive results), even when treatment has effectively cleared the patient of parasites [30]. It is therefore possible that some clinicians withheld prescription of anti-malarials to patients with a positive RDT on grounds that a patient previously treated for malaria could have presented with an alternative febrile illness and a false positive RDT result. Testing the validity of this hypothesis was not feasible, as the number of patients with a positive result but not prescribed an anti-malarial was small.

Overall, results of this study point to a significant improvement in HCW adherence levels to the test and treat component of guidelines, a trend that could be explained by growing HCW confidence in test results, likely an outcome of years of sustained training, mentorship, and supervision of HCW on malaria case management [31, 32]. Recent studies from Angola [18, 33], Guinea [34], Kenya [19, 35, 36], Malawi [37], and Zambia [38–40] point to improved HCW adherence to test and treat policy. However, in other countries, like Mozambique [31, 41], Madagascar [42], Congo [43], Cameroon [44], and Mali [45] gains have been less impressive.

Overall, performance levels remained relatively high, even when the outcome was based on a composite indicator; appropriate malaria case management encompassing testing and adherence to treatment as per national guidelines. Performance levels were comparable across facility levels, types and age categories, with the exception of PFP facilities where only 50.7% (Table 2) of patients received appropriate malaria case management. Problems of PFP in Uganda have been described before including low testing rates [24] and use of non-recommended anti-malarials as first line treatment of uncomplicated malaria [46]; practices consistent with findings of this study. Prescription of non-recommended artemisinin-based combinations, such as DP, though a deviation from policy recommendation may be an acceptable practice in certain circumstances. However, even when appropriate case management was defined based on any artemisinin-based combination prescribed; a more accommodative treatment outcome, adherence levels at PFP facilities increased (58.8%) but still remained sub-optimal. This implies that lack of testing was the primary barrier to providing appropriate care at PFPs, as has been demonstrated in other studies [31, 32]. Testing is a key step in the cascade of events that culminates in appropriate malaria case management [47]. Once the testing step is breached, a ripple effect of failure spreads through the cascade, with presumptive treatment of malaria and lack of adherence to test results promoted. Among those not tested, presumptive treatment of malaria was common at PFP and PNFP facilities, potentially explaining why PNFP facilities were also less likely to provide appropriate malaria case management. Causing change in the private sector remains an uphill task, requiring novel and innovative strategies beyond what has been tried before [48, 49]. Efforts must be cross-cutting and sustainable, promoting understanding of benefits of adherence to malaria case management guidelines by providers and also clients. Regulation and oversight of the private sector remains paramount, as the desire to profit from patient visits often conflicts with adherence to guidelines [25, 50]. Non-coercive methods of regulation including training, supervision and provision of guidelines, factors that were associated with appropriate malarial care in this study (Table 3), should be considered as strategies for improving performance at PFPs. HC III and IIs were more likely to provide appropriate malaria case management compared to HC IVs, a fact likely attributed to low patient numbers seen at these facilities. Low patient numbers potentially translates to (1) high quality HCW-patient interactions and (2) lower opportunities of stock out of essential commodities, explaining better services at HCIII and IIs. These facts may also explain the observed higher patient satisfaction scores at HC III and IIs.

Compared to patients prescribed AL at PNFP and PFP facilities, patients seen at GOU facilities were less likely to have received AL at the facility. This fact is likely attributed to the higher prevalence of AL stock-outs at GOU facilities. AL stock-out at GOU facilities is a problem that undermines effectiveness of malaria case management in two ways. First, patients eligible for treatment with AL may not be able to afford the medicine. Second, failure to get medicines at the facility frustrates patients, promoting alternative care-seeking patterns for subsequent illness episodes, potentially delaying access to appropriate care [24, 51]. While strengthening of the national supply chain has reduced the occurrence of stock-outs in public facilities, pockets of AL stock-outs persist [52]. Isolated cases of AL stock-outs point to a mismatch between supply and demand, attributed to poor forecasting or misuse of medicines at the facility. Directly observed treatment (DOT) of AL was infrequent at all facilities and this can be explained by non-prioritization of this aspect of malaria case management during trainings. Inadequate staffing levels at the pharmacy department further compounds this problem, especially in large volume sites, where patient numbers can be overwhelming. Indeed, only half of all HC IVs had a dispenser on site with each facility having one dispenser, and only nine and one of all HC IIIs and HC IIs had a dispenser. Nevertheless, many patients (71.4%) were advised to complete the dose and take medicines after a meal, information which may have been easy to relay to patients as they got their medicines. However, details on how to respond after an adverse event or vomiting the medicines were seldom discussed. Future training programs should emphasize the importance of dispensing and post dispensing standards as these promote adherence to treatment at home.

This study had some limitations. Only a sub-set of PFP facilities were studied; those registered and recognized by the MOH, with concern that standards at non-registered PFPs, the majority and more likely to falter on standards, were not represented in the study sample. Additionally, the small number of PFP facilities studied undermining the power of the study to detect some statistically significant findings in relation to PFPs. Not all aspects contributing to effective malaria case management including time to seeking care, appropriateness of AL dosing, and adherence to treatment were studied. Consequently, some factors that impact on the effectiveness of malaria case management services were not accounted for. HCW interviews were self-administered; therefore, the opportunity to validate responses to the non-test questions was missed. To mitigate this problem, questions related to knowledge and experiences were clearly framed maximizing accuracy of responses. Importantly, related facility and HCW data yielded similar results suggesting that HCW responses were valid. Nonetheless, despite all limitations, the study findings remain pertinent, providing an up-to-date account of the status of malaria case management practise in the region.

## Conclusion

Overall, there is improvement in adherence to malaria case management guidelines by HCWs in the Busoga sub-region, with gaps noted in both the public and private sector. Absence of AL at public facilities resulted into a substantial proportion of eligible patients not receiving treatment at the facility undermining effectiveness of care, particular if patients can’t afford to buy medicines. To maximize levels of adherence to guidelines at the public sector, the supply chain of essential commodities relevant to malaria case management, such as AL, needs to be tightened. Compared to centralized solutions, district level solutions could provide a more robust mechanism to addressing unanticipated AL stock-outs at facilities. Additionally, the study findings lend credence to the importance of availing all health care workers with guidelines relevant to malaria case management promoting adherence to guidelines. Effective supervision systems for HCW at both public and private facilities should be emphasized as these realign practice to standards [53]. For the private sector, challenges are unique, driven by lack of knowledge, the desire to profit, and lack of incentive to adhere to guidelines calling for regulation and innovative and sustainable strategies that promote adherence to guidelines.

## Supplementary Information

**Additional file 1: **The health facility questionnaire.

**Additional file 2: **The health care worker questionnaire.

**Additional file 3: **The patient exit interview questionnaire. The questionnaire was transformed into an electonic version enabling collection of data using tablets.

**Additional file 4: **A tabular presentation of data used to determine the weights to consider when adjusting sampled facilities to match the population distribution of facilities. For the final analysis weights presented in Table 1 were considered.

**Additional file 5: **A figure of the study profile showing the number of patients screened, number of patients excluded, reason for exclusion, and the final number of patients enrolled for patient exit interviews (PEI).

**Additional file 6: **A figure of the study profile for inlcuding facilities where all aspects of the study; HFAs PEI and HCW interviews were conducted. The following information is included: (a) The number of facilites enrolled for HFAs, PEIs, and HCW interviews, respectively; (b) The number of facilites where HFAs, PEIs, and HCW interviews were conducted but excluded for missing data on any one of  three (HFA, PEI and HCW) aspects of the study; (c) The number of facilites (with the corresponding numbers of PEIs or HCW interviews) where all three aspects of the study were conducted and therefore included in the final analysis.

**Additional file 7:** A tabular presentation of how facilities were sampled, enrolled, replaced (new) or excluded arriving at the total number of facilities  included  in the final analysis by district, overall and stratified by facility type (level and owner).

**Additional file 8:** A table showing HCW interview response rates stratified by staff position and grouped into HCWs targeted (directly involved in providing patient care) and those not targeted (indirectly involved in providing patient care) for interviews.

**Additional file 9:** Generalized estimating equation (GEE) model was used to identify independent patient, facility, and health care worker factors associated with appropriate malaria case management defined in three different ways based on varying  treatment options for confirmed malaria: (a) AL prescribed, (b) ACT prescribed, or (c) AL given to the patient.

## Data Availability

The datasets used and/or analysed during the current study are available from the corresponding author on reasonable request.
