# Supplementary material for: Adherence to malaria management guidelines by health care workers in the Busoga sub-region, eastern Uganda
Source: Malar J. 2022 Jan 25;21:25. doi: 10.1186/s12936-022-04048-2 (PMC8788114; doi:10.1186/s12936-022-04048-2)
Supplement: Supplementary file 1 — Additional file 1: The health facility questionnaire. [file 12936_2022_4048_MOESM1_ESM.pdf]

Additional file 1: Health facility assessment questionnaire

<table>
<tr><td>Name of facility code</td><td colspan="7"></td></tr>
<tr><td>Level</td><td>□ NRH</td><td>□ RRH</td><td>□ RH</td><td>□ GH</td><td>□ HCIV</td><td>□ HCIII</td><td>□ HCII</td></tr>
<tr><td>Ownership</td><td>□ GOU</td><td>□PNFP</td><td>□ PFP</td><td colspan="4">□ Community</td></tr>
<tr><td rowspan="3">Authority</td><td>□ AIC</td><td>□ CAFU</td><td>□ CBO</td><td>□ MOES</td><td>□ MOH</td><td colspan="2">□ NGO</td></tr>
<tr><td>□ Private</td><td>□ SDA</td><td>□ SOS</td><td>□ TASO</td><td>□ UCBHCA</td><td colspan="2">□ UCMB</td></tr>
<tr><td>□ UMMB</td><td>□ UNHCR</td><td>□ UPDF</td><td>□ UOBM</td><td>□ UPF</td><td>□ UPMB</td><td>□ UPS</td></tr>
<tr><td>Facility code</td><td>|____|____| ____|</td><td>Subcounty code</td><td>|____|____| ____|</td><td>District code</td><td colspan="3">|____|____|</td></tr>
<tr><td>Unique ID</td><td colspan="7">Facility code #: |____|____| ____| Subcounty code: |____|____| ____| District Code |____|____|</td></tr>
<tr><td>Location</td><td colspan="3">□ Urban</td><td colspan="4">□ Rural</td></tr>
<tr><td>Date of assessment</td><td colspan="7">day|_____|_____| month|_____|_____| year |_____|_____|</td></tr>
<tr><td>Interview time</td><td colspan="7">|_____|_____| hr |_____|_____| min</td></tr>
<tr><td>Name of interviewer 1</td><td colspan="7"></td></tr>
<tr><td>Name of interviewer 2</td><td colspan="7"></td></tr>
<tr><td>Facility code</td><td colspan="7">Facility Code: |____|____| ____| Sub County Code: |____|____| ____| District Code |____|____| ____|</td></tr>
<tr><td>GPS coordinates</td><td colspan="7"></td></tr>
</table>

GENERAL INFORMATION

FIND THE MANAGER, THE PERSON IN-CHARGE OF THE FACILITY, OR MOST SENIOR HEALTH WORKER RESPONSIBLE FOR OUTPATIENT SERVICES WHO IS PRESENT AT THE FACILITY. READ THE FOLLOWING GREETING:

Good day! My name is _____________________. We are here on behalf of [IMPLEMENTING AGENCY] conducting a survey of health facilities to assist the government in knowing more about health services in [COUNTRY].

Now I will read a statement explaining the study.

Your facility was selected to participate in this study. We will be asking you questions about various health services. Information about your facility may be used by the [MOH], organizations supporting services in your facility, and researchers, for planning service improvement or for conducting further studies of health services.

Neither your name nor that of any other health worker respondents participating in this study will be included in the dataset or in any report; however, there is a small chance that any of these respondents may be identified later. Still, we are asking for your help to ensure that the information we collect is accurate.

You may refuse to answer any question or choose to stop the interview at any time. However, we hope you will answer the questions, which will benefit the services you provide and the nation.

If there are questions for which someone else is the most appropriate person to provide the information, we would appreciate if you introduce us to that person to help us collect that information.

At this point, do you have any questions about the study? Do I have your agreement to proceed?

<table>
<tr><td>May I begin the interview?</td><td>□ Yes</td><td>□ No</td></tr>
<tr><td>Interview start time</td><td colspan="2">|_____|_____| hr |_____|_____| min</td></tr>
</table>

**STAFFING**					
I have a few questions on staffing for this facility. Please tell me how many staff with each of the following qualifications are currently assigned to, employed by, or seconded to this facility. Please count each staff member only once, on the basis of the highest technical or professional qualification. For doctors, I would also like to know, of the total number, how many are part-time in this facility.					
Cadre	Number				
	Employed	Volunteer	On site	Study leave	AWOL
General non specialist medical doctor (Degree in medicine)					
Specialist medical doctor (Masters Degree in sub-speciality)					
Senior Medical Officer					
Medical Officer					
Senior Medical Clinical Officer					
Ophthalmic Clinical Officer					
Medical Clinical Officer					
Clinical Officer (Speech Therapy)					
Senior Nursing Officer					
Public Health Nurse					
Nursing Officer – Midwifery (Assistant)					
Nursing Officer – Nursing (Assistant)					
Nursing Officer - Psychiatry					
Enrolled Midwife					
Enrolled Nurse					
Enrolled Nurse - Psychiatry					
Public Health Dental Officer					
Medical Laboratory Technician					
Medical Laboratory Assistant					
Medical Records Officer					
Health Information Assistant					
Dispenser					
Health Inspector					
Radiographer					
Vector Control Officer					
Anaesthetic officer					
Assistant Entomological Officer					
Assistant Health Educator					
Theatre Assistant					
Accounts Assistant					
Anaesthetic Assistant					
Health Assistant					
Stores Assistant					
Cold Chain Assistant					
Office Typist					
Car Driver					
Nursing Assistant					
Askari (Security Guard)					
Porter					

Employed: On pay roll: Volunteer: Qualified, is officially on duty (presence on site is known by facility I/C: Onsite: Is the member of staff on site on the day of the assessment: AWOL: Has absconded un-officially from facility

INFRASTRUCTURE				
Does this facility have?	Functional land line (that is available to call outside at all times)		□Yes	□ No
	Functional cellular phone or private phone (supported by the facility)		□Yes	□ No
	Functional short-wave radio or radio calls		□ Yes	□ No
	Functioning computer		□ Yes	□ No
	Access to email or internet today (wireless or wired)		□ Yes	□ No
	Ambulance or transport for emergencies		□ Yes	□ No
**AMBULANCE TRANSPORT FOR EMERGENCIES**				
Does this facility have a functional ambulance (or other vehicle) stationed at the facility			□ Yes	□ No
**If yes,**	Is fuel for the ambulance/other vehicle available today?		□ Yes	□ No
	Is the driver for the ambulance/other vehicle available today?		□ Yes	□ No
	Do clients contribute to pay for fuel		□ Yes	□ No
Does this facility have access to a functional ambulance/ other vehicle stationed elsewhere		□ Yes	□ No	□ NA
**If yes,**	Is fuel for the ambulance/other vehicle available today?		□ Yes	□ No
	Is the driver for the ambulance/other vehicle available today?		□ Yes	□ No
	Do clients contribute to pay for fuel		□ Yes	□ No
**POWER SUPPLY**				
Does your facility have power from any source			□ Yes	□ No
What is the facility main source of electricity	□ National or Community Grid	□ Generator		
	□ Solar	□ Wind		
What is electricity used for at the facility	□ Stand-alone Electrical Medical Devices			
	□ Lighting and Communication			
	□ Stand-alone Electrical Medical Devices			
	□ Standalone Electrical Medical Devices, Lighting and Communication			
Does this facility a power back up	□ Yes	□ No		
If yes, what is the source	□ National or Community Grid	□ Generator		
	□ Solar	□ Wind		
During the past 7 days was electric available at all times from the main or back up source	□ Always available			
	□ Often available *(interruptions of < 2 hours less than 3 days a week)*			
	□ Sometime available (frequent or prolonged (>2hrs) day			
If the facility has a generator,	Is it functional		□ Yes	□ No
	Is there fuel		□ Yes	□ No
	Is there a charged battery	□ Yes	□ No	□ NA
If the facility has a solar panel generator, status	□ Functional	□ Partial (Batt need service/change)		
	□ Not functional	□ Don't know		

BASIC CLIENT AMENITIES			
On average, how many hours per day is this facility open	□ 4 hours or less		□ 8 hours
	□ 9 to 16 hours	□ 17 to 23 hours	□ 24 hours
What is the **most commonly used** source of water?	□ Piped into facility	□ Protected dug well	□ unprotected dug well
	□ Protected spring	□ Unprotected spring	□ Rainwater collection
	□ Bottled water	□ Cart/Drum	□ Tanker truck
	□ Surface water	□ Don't know	□ No source

Is water available from the **commonly used** source (observe for availability of water?	□ Yes	□ No

SUPERVISION		
Has this facility received a supervision visit from the higher level this year (2020) or the in 2019	□ Yes	□ No
If yes, specify the month and year	**day|_____|_____| month|_____|_____| year |_____|_____|**	
If yes, did the supervisor assess the following service points	□ Pharmacy	□ Staffing
	□ Data	□ Laboratory
If yes, were the following topics/areas covered or discussed	□ Uncomplicated malaria case management	
	□ Severe malaria case management	
	□ Malaria microscopy	
	□ Malaria RDT	

BASIC EQUIPMENT

Please tell me if the following basic equipment and supplies used in the client services are available and function in this facility today (OP and IP department)

ASK TO SEE THE ITEMS

**Item**	**Available**		**Functioning**	
Adult weighing scale	□ Yes	□ No	□ Yes	□ No
Child weighing scale	□ Yes	□ No	□ Yes	□ No
Infant weighing scale	□ Yes	□ No	□ Yes	□ No
Stadiometer	□ Yes	□ No	□ Yes	□ No
Thermometer	□ Yes	□ No	□ Yes	□ No
Blood pressure	□ Yes	□ No	□ Yes	□ No
Light source	□ Yes	□ No	□ Yes	□ No
Intravenous infusion set	□ Yes	□ No	□ Yes	□ No
Blood transfusion set	□ Yes	□ No	□ Yes	□ No
IV cannula	□ Yes	□ No	□ Yes	□ No
Oxygen (concentrator)	□ Yes	□ No	□ Yes	□ No
Oxygen (tank)	□ Yes	□ No	□ Yes	□ No
Oxygen (piped)	□ Yes	□ No	□ Yes	□ No
Nasal prongs/Nasal mask	□ Yes	□ No	□ Yes	□ No
Flow meter for Oxygen therapy	□ Yes	□ No	□ Yes	□ No
Resuscitation table	□ Yes	□ No	□ Yes	□ No
Suction pump (manual)	□ Yes	□ No	□ Yes	□ No
Suction pump (electric)	□ Yes	□ No	□ Yes	□ No
Suction catheter	□ Yes	□ No	□ Yes	□ No
Nasogastric Tube	□ Yes	□ No	□ Yes	□ No

**INFECTION CONTROL PRECAUTIONS: Please** tell me if the following resources/supplies used for infection prevention are available in the **general OP/IP** area of this facility today<br>(**Tick where applicable**) **O=Observed, R: Reported not seen; NA: Not available**							
**Item**	**O**	**R**	**NA**	**Item**	**O**	**R**	**NA**
Clean running water (flowing and tap)	□	□	□	Washing point	□	□	□
Hand washing soap	□	□	□	Goggles	□	□	□
Hand washing facility	□	□	□	Gloves	□	□	□
Alcohol (70%) based hand rub	□	□	□	Masks	□	□	□
Disposable gloves	□	□	□	Health workers wearing mask	□	□	□
Sterile gloves	□	□	□	Disposable syringe and needles	□	□	□
Waste receptacle	□	□	□	Auto disposable syringes	□	□	□
Sharps containers	□	□	□	JIK (Chlorine) disinfectant	□	□	□

**SCREENING**		
Is there a screening tent at the entrance of the facility	□ Yes	□ No
If yes, is there a health worker actively screening patient	□ Yes	□ No
If yes, is there a health worker taking temperature of patients	□ Yes	□ No
**TRIAGE**		
Is there a triage desk at the OP/Waiting area	□ Yes	□ No
If yes, is there a health worker actively triaging patient	□ Yes	□ No
**GUIDELINES AND WALL CHARTS (MALARIA)**		
Is there a facility copy of 2014 malaria guideline for HWs?	□ Yes	□ No
Is there a facility copy of IMCI guideline for HWs?	□ Yes	□ No
Is there a facility copy of Uganda Clinical Guidelines 2016?	□ Yes	□ No
**Are the following malaria wall charts exposed at the facility**		
Algorithm for assessing and treating children <5 yrs with fever? (Y/N)	□ Yes	□ No
AL dispensing procedure and dosing schedule? (Y/N)	□ Yes	□ No
Malaria outpatient algorithm for older children and adults?	□ Yes	□ No
How to Do the Rapid Malaria Test for Malaria	□ Yes	□ No
Malaria outpatient algorithm for children and adults (**new chart**)? (Y/N)	□ Yes	□ No
Artesunate IV/IM administration poster? (Y/N)	□ Yes	□ No
**GUIDELINES AND WALL CHARTS (COVID-19)**		
Is there a facility copy of 2020 National Guidelines for management of COVID-19	□ Yes	□ No
Is there a facility copy of 2020 Guidelines for mask use	□ Yes	□ No
Is there a copy of 2020 Guidance on Continuity of Essential Health Services during the COVID-19 outbreak	□ Yes	□ No
**Are the following COVID-19 wall charts exposed at the facility**		

Hand washing	□ Yes	□ No	Respiratory Hygiene	□ Yes	□ No
Mode of spread	□ Yes	□ No	Referral	□ Yes	□ No
Symptoms and Signs	□ Yes	□ No	Screening	□ Yes	□ No

RELEVANT TRAINING

CASE MANAGEMENT

Level of cadre	In service training on Malaria Case Management					In service training in malaria RDT					In service training COVID-19			
											Prevention	Signs Symptoms	Case Definition	Diagnosis
	2020	2019	2018	2017	2016	2020	2019	2018	2017	2016				
General non specialist medical doctor														
Specialist medical doctor														
Clinical Officers														
Nurse														
Enrolled Nurses														
Enrolled midwifes														
Registered nurses														
Registered midwifes														
Comprehensive nurses														
Nursing assistants														

LABORATORY STAFFING AND RELEVANT IN-SERVICE TRAINING

Level	In service training in the past years										In service training COVID-19			
	microscopy					RDT					Prevention	Signs Symptoms	Case Definition	Diagnosis
	2020	2019	2018	2017	2016	2020	2019	2018	2017	2016				
Sen Laboratory Technologists														
Laboratory Technologists														
Sen Laboratory Technicians														
Laboratory Technicians														
Laboratory Assistants														

DRUG DISPENSING STAFFING AND IN-SERVICE TRAINING

Cadre	In-service training on management of malaria medicines					In service training COVID-19			
	2020	2019	2018	2017	2016	Prevention	Signs Symptoms	Case Definition	Diagnosis
Pharmacist									
Senior Dispenser									
Dispenser									

AVAILABILITY AND QUALITY OF MALARIA DIAGNOSTIC SERVICES			
Is malaria microscopy routinely provided at health facility?		□ Yes	□ No
**If YES**, is **malaria microscopy** service functional **today**		□ Yes	□ No
If **NOT** functioning, what is the main reason?	□ Microscope spoilt	□ Stains and other supplies out of stock	□ Power outage
	□ Lab tech not present?	□ Management decision	□ Other (specify)? (Y/N)
Which blood smear is routinely prepared on single slide?	□ Only thick smear	□ Only thin smear	□ Both
Which staining solution for malaria smears is routinely used?	□ Giemsa	□ Field stain	
Are parasite species routinely reported? (Y/N) [check register]		□ Yes	□ No
Is parasite count routinely reported?		□ Yes	□ No
If Yes, which reporting method	□ Plus system	□ Scanty/moderate/heavy	
	□ Number of parasites per microliter/ WBC	□ Number of parasites per high power field	
	□ Other, specify, ______________________________		
Are the national guidelines for implementation of parasite-based diagnosis of malaria in Uganda available?		□ Yes	□ No
**Does laboratory have 8 SOPs for malaria parasitology?**	Collection and preparation of thick and thin blood smears?	□ Yes	□ No
	Preparation of buffered water?	□ Yes	□ No
	Preparation of Giemsa stock solution?	□ Yes	□ No
	Staining of thick and thin smears using Giemsa stain?	□ Yes	□ No
	Reading and reporting malaria parasites on stained smears?	□ Yes	□ No
	Cleaning and storage of slides?	□ Yes	□ No
	Use, care and maintenance of microscopy?	□ Yes	□ No
	Rapid diagnostic tests?	□ Yes	□ No
Do power cuts interfere with the ability to perform malaria microscopy?		□ Yes	□ No
If Yes, how many days in average in a week? (estimate number of days)			

AVAILABILITY OF LABORATORY SUPPLIES				
	**In stock today?**		**Out of stock 7 or more consecutive days in past 3 months**	
**Availability of microscopy supplies?**	□ Yes	□ No	□ Yes	□ No
Immersion oil?	□ Yes	□ No	□ Yes	□ No
Lancets?	□ Yes	□ No	□ Yes	□ No
Methanol?	□ Yes	□ No	□ Yes	□ No
Field stain	□ Yes	□ No	□ Yes	□ No
Leishman	□ Yes	□ No	□ Yes	□ No
Giemsa stain	□ Yes	□ No	□ Yes	□ No
Availability of malaria RDTs today?	□ Yes	□ No	□ Yes	□ No

If yes,	Type 1: Name		Expiry date	**day|___|___| mo|___|___| yr |___|___|**
	Type 2: Name		Expiry date	**day|___|___| mo|___|___| yr |___|___|**

Does the laboratory participate in a malaria EQA scheme for microscopy?	□ Yes	□ No
If yes, are results from EQA exercises recorded in a register?	□ Yes	□ No
If yes, are slides stored for re-reading?	□ Yes	□ No
If yes, does the laboratory receive feedback for its EQA schemes	□ Yes	□ No
If yes, are positive control slides available for testing new batches of Field/Giemsa stain?	□ Yes	□ No
Does the laboratory participate in a malaria EQA scheme for RDT?	□ Yes	□ No

AVAILABILITY OF AL AND MALARIA MEDICINES INVENTORY MATERIALS ON SURVEY DAY

	Non-expired quantity [physical count]			Non-expired quantity [record count]			Expired quantity [physical count]
**1st line for uncomplicated malaria**	Store	Dispensing area	Total	Stock card	AL register	Total	
AL 6 pack [No of blisters]							
AL 12 pack [No of blisters]							
AL 18 pack [No of blisters]							
AL 24 pack [No of blisters]							
**1st line severe malaria**							
IV Artesunate [No of Vials]							
Rectal Artesunate [No of packs]							

**Other line**	**Quantity**	
	**Expired**	**None expired**
Artemether-Amodiaquine		
Dihydroartemesinin-Piperaquine (DP)		
Quinine injections [No of vials]		
Chloroquine tablets [No of tablets]		
Chloroquine syrup [No of liters]		
Chloroquine injections [No of vials]		
SP tablets [No of tablets]		
SP syrup or drops [No of bottles]		
Amodiaquine tablets [No of tablets]		
Amodiaquine syrup [No of liters]		
Quinine tablets [No of tablets]		
Other types, name them		
Other types, name them		

STOCKING AND ORDERING OF ANTIMALARIALS		
Is drug stock/bin card available at HF? (Y/N)	□ Yes	□ No
If Yes, is it regularly updated [check for last one month]?	□ Yes	□ No
Is dispenser's log available at HF? (Y/N)	□ Yes	□ No
If Yes, is it regularly updated [check for last one month]?	□ Yes	□ No
Is monthly summary form for malaria medicines available at HF	□ Yes	□ No

Name of regular medicine supplier	□ NMS	□ JMS	□ Other, specify

Date of last AL delivery to the health facility? [check delivery note]	**day|____|_____| mo|_____|_____| yr |_____|_____|**

Does the facility function on **pull** (ordering) system - ordering AL	□ Yes	□ No

If yes,		Quantity ordered? (number)	Quantity received? (Number)		Quantity ordered? (number)	Quantity received? (Number)
	AL 6 tabs pack			AL 18 tabs pack		
	AL 12 tabs pack			AL 24 tabs pack		

Date of last AL delivery preceding last delivery [check delivery note]	**day|____|_____| mo|_____|_____| yr |_____|_____|**

Does the facility function on **push** (ordering) system - ordering AL	□ Yes	□ No

If yes,		Quantity ordered? (number)	Quantity received? (Number)		Quantity ordered? (number)	Quantity received? (Number)
	AL 6 tabs pack			AL 18 tabs pack		
	AL 12 tabs pack			AL 24 tabs pack		

**Date of AL delivery** preceding last AL delivery? [check delivery note]	**day|____|_____| mo|_____|_____| yr |_____|_____|**

Availability of other NON-EXPIRED medicines in STORE on survey day								
Cotrimoxazol tab	□ Y	□ N	Kanamycin injection	□ Y	□ N	Diazepam inj	□ Y	□ N
Cotrimoxazol syrup	□ Y	□ N	Procaine penicillin injection	□ Y	□ N	Phenobarbitone inj	□ Y	□ N
Amoxycillin capsules	□ Y	□ N	Tetracycline Eye Ointment	□ Y	□ N	Phenobarbitone tab	□ Y	□ N
Amoxycillin syrup	□ Y	□ N	Paracetamol tab	□ Y	□ N	Iron tablets	□ Y	□ N
Ceftriaxone injection	□ Y	□ N	Adrenaline inj	□ Y	□ N	**Dextrose 5%**	□ Y	□ N
Ciprofloxacin tablets	□ Y	□ N	ORS Sachets	□ Y	□ N	Dextrose 50%	□ Y	□ N
Erythromycin tablets	□ Y	□ N	Kanamycin injection	□ Y	□ N	Albendazole tab	□ Y	□ N
Chloramphenicol caps	□ Y	□ N	Chloramphenicol injection	□ Y	□ N	Gentamycin injection	□ Y	□ N
Chloramphenicol syrup	□ Y	□ N	Benzylpenicillin injection	□ Y	□ N	Hydrocortisone inj	□ Y	□ N
Clotrimazole cream	□ Y	□ N	Zinc Sulphate	□ Y	□ N	Magnesium sulphate	□ Y	□ N

Other, specify	□ Y	□ N	Other, specify	□ Y	□ N

ANTIMALARIAL STOCK-OUTs in Mar, Apr, May 2020

[for each non-expired AL preparation, tick the **day out of stock** in each evaluation month and calculate **Total number of stock-out days** over 3 months period; if stock-out day information is not available from AL record books enter NA in the box and **do not count** total number of stock-out days]**.**

AL 6 tablet pack	1	2	3	4	5	6	7	8	9	10	11	12	13	14	15	16	17	18	19	20	21	22	23	24	25	26	27	28	29	30	31	Total
Mar 2020																																
Apr 2020																																
May 2020																																
AL 12 tablet pack	1	2	3	4	5	6	7	8	9	10	11	12	13	14	15	16	17	18	19	20	21	22	23	24	25	26	27	28	29	30	31	Total
Mar 2020																																
Apr 2020																																
May 2020																																
AL 18 tablet pack	1	2	3	4	5	6	7	8	9	10	11	12	13	14	15	16	17	18	19	20	21	22	23	24	25	26	27	28	29	30	31	Total
Mar 2020																																
Apr 2020																																
May 2020																																
AL 24 tablet pack	1	2	3	4	5	6	7	8	9	10	11	12	13	14	15	16	17	18	19	20	21	22	23	24	25	26	27	28	29	30	31	Total
Mar 2020																																
Apr 2020																																
May 2020																																
SP tablets	1	2	3	4	5	6	7	8	9	10	11	12	13	14	15	16	17	18	19	20	21	22	23	24	25	26	27	28	29	30	31	Total
Mar 2020																																
Apr 2020																																
May 2020																																
QN tablets	1	2	3	4	5	6	7	8	9	10	11	12	13	14	15	16	17	18	19	20	21	22	23	24	25	26	27	28	29	30	31	Total
Mar 2020																																
Apr 2020																																
May 2020																																
QN injection	1	2	3	4	5	6	7	8	9	10	11	12	13	14	15	16	17	18	19	20	21	22	23	24	25	26	27	28	29	30	31	Total
Mar 2020																																
Apr 2020																																
May 2020																																
Artesunate inj	1	2	3	4	5	6	7	8	9	10	11	12	13	14	15	16	17	18	19	20	21	22	23	24	25	26	27	28	29	30	31	Total
Mar 2020																																
Apr 2020																																
May 2020																																

ABSENCE OF MALARIA MICROSCOPY SERVICE AND RDT STOCK-OUT IN MARCH, APRIL, MAY 2020

[for each evaluation day, tick the box if malaria microscopy service was not provided or RDTs were out of stock and calculate Total number of absence or stock-out days over the whole 3 months period; if stock-out day information is not available from laboratory books enter NA in the box an do not count total number of stock-out days]

Malaria microscopy absent	1	2	3	4	5	6	7	8	9	10	11	12	13	14	15	16	17	18	19	20	21	22	23	24	25	26	27	28	29	30	31	Total
Mar 2020																																
Apr 2020																																
May 2020																																
**Malaria RDT stock-out**	**1**	**2**	**3**	**4**	**5**	**6**	**7**	**8**	**9**	**10**	**11**	**12**	**13**	**14**	**15**	**16**	**17**	**18**	**19**	**20**	**21**	**22**	**23**	**24**	**25**	**26**	**27**	**28**	**29**	**30**	**31**	**Total**
Mar 2020																																
Apr 2020																																
May 2020																																
**Both diagnostics absent**	**1**	**2**	**3**	**4**	**5**	**6**	**7**	**8**	**9**	**10**	**11**	**12**	**13**	**14**	**15**	**16**	**17**	**18**	**19**	**20**	**21**	**22**	**23**	**24**	**25**	**26**	**27**	**28**	**29**	**30**	**31**	**Total**
Mar 2020																																
Apr 2020																																
May 2020																																

STOCK OUT OF ANTIMALARIAL DRUGS AND ABSENCE OF DIAGNOSTICS FOR 7 CONSECUTIVE DAYS IN MARCH, APRIL, MAY 2020

Antimalarial	According to record review? (Y/N/NA)			If Not available (NA) in records, according to health worker's report? (Y/N/DK)		
Artemether-lumefantrine 6 tablets pack	□ Yes	□ No	□ N/A	□ Yes	□ No	□ N/A
Artemether-lumefantrine 12 tablets pack	□ Yes	□ No	□ N/A	□ Yes	□ No	□ N/A
Artemether-lumefantrine 18 tablets pack	□ Yes	□ No	□ N/A	□ Yes	□ No	□ N/A
Artemether-lumefantrine 24 tablets pack	□ Yes	□ No	□ N/A	□ Yes	□ No	□ N/A
All four Artemether-lumefantrine packs	□ Yes	□ No	□ N/A	□ Yes	□ No	□ N/A
SP tablets	□ Yes	□ No	□ N/A	□ Yes	□ No	□ N/A
Quinine tablets	□ Yes	□ No	□ N/A	□ Yes	□ No	□ N/A
Quinine injections	□ Yes	□ No	□ N/A	□ Yes	□ No	□ N/A
Artesunate injection	□ Yes	□ No	□ N/A	□ Yes	□ No	□ N/A
Malaria RDT	□ Yes	□ No	□ N/A	□ Yes	□ No	□ N/A
Malaria microscopy	□ Yes	□ No	□ N/A	□ Yes	□ No	□ N/A

AVAILABILITY OF HMIS FORMS			
Are these HMIS forms/registers available at the facility	HMIS Form 015: Stock card	□Yes	□ No
	HMIS Form 016: Daily drug dispensing log	□Yes	□ No
	HMIS Form 031: Outpatient Register	□ Yes	□ No
	HMIS Form 055: Laboratory Register	□Yes	□ No
	HMIS Form 033b Health Unit Weekly Epidemiological Surveillance Report	□ Yes	□ No
	HMIS Form 105: Health Unit Monthly Outpatient Report	□ Yes	□ No
	HMIS Form 108: Health Unit Inpatient Register	□ Yes	□ No

TIMELINESS OF SUBMISSION OF REPORTS									
HMIS FORM	TIME PERIOD		Due date for report	Date of Submission [c]	Interval [d]	Submitted on time			
HMIS Form 033b Health Unit Weekly Epidemiological Surveillance Report	Week[a]/Year	Week 24 2020	08/06/2020			□Yes	□ No	□ Not submitted	□ Not available
		Week 25 2020	15/06/2020						
		Week 26 2020	22/06/2020						
		Week 27 2020	29/06/2020						
		Week 28 2020	06/07/2020						
HMIS Form 105: Health Unit Monthly Outpatient Report	Month[b]/Year	April 2020	07/05/2020						
		May 2020	07/06/2020						
		June 2020	07/07/2020						

[a]Ask to see specific weekly reports and ask the date they were submitted.
[b]As to see specific monthly report and record the date of submission as indicated on the report.
[c]If not submitted leave the date of submission blank
[d]Interval is the difference between date of submission and due date of report
