# Supplementary material for: Adherence to malaria management guidelines by health care workers in the Busoga sub-region, eastern Uganda
Source: Malar J. 2022 Jan 25;21:25. doi: 10.1186/s12936-022-04048-2 (PMC8788114; doi:10.1186/s12936-022-04048-2)
Supplement: Supplementary file 2 — Additional file 2: The health care worker questionnaire. [file 12936_2022_4048_MOESM2_ESM.pdf]

Additional file 2: Health care worker survey questionnaire

**Name of facility**							
**Level**	□ NRH	□ RRH	□ RH	□ GH	□ HCIV	□ HCIII	□ HCII	□ HCI
**Ownership**	□ GOU	□PNFP	□ PFP	□ Community			
**Health worker #**		**Facility code**		**District code**			
**Date of completion**	day|_____|_____| month|_____|_____| year |_____|_____|						
**Unique ID**	Health worker #: |____|____| ____| Facility Code: |____|____| ____| Must be same as above District Code |____|____| ____| Must be same as above						

**GENERAL INFORMATION ABOUT THE HEALTH WORKER**				
What is your age?		What is your sex	□ Male	□Female
What is the health worker' position?	□ General medical doctor	□ Specialist medical doctor	□ Clinical Officer	□ Nurse (Degree)
	□ Registered nurse	□ Registered midwifes	□ Enrolled Nurse	□ Enrolled midwifes
	□ Nursing aid	□ Other, specify		
Are you the facility in-charge?		□ Yes	□ No	

**IN-SERVICE TRAINING RELATED TO MALARIA**			
**Have you ever attended IMCI training? (in the last five years)**		□ Yes	□ No
If Yes,	Date of training? (month-year)	day|____|____| month|____|____| year |____|____|	
	Was use of Artemether Lumefantrine part of the IMCI course?		
	Was use of RDT part of the IMCI course?		
**Have you attended malaria case management training that included AL use (in the last five years)**		□ Yes	□ No
If yes	Date	day|____|____| month|____|____| year |____|____|	
	Organization giving the course (name)		
	Duration (in days)		
	Clinical practice included?	□ Yes	□ No
	Was use of RDTs part of the course?	□ Yes	□ No
**Have you ever attended RDT specific malaria training?**		□ Yes	□ No
**If Yes**, **date** of training?	**day|____|____| month|____|____| year |____|____|**		
**Have you ever been trained/oriented how to use injectable Artesunate?**		□ Yes	□ No
If yes, date of training	**day|____|____| month|____|____| year |____|____|**		
**Have you ever been trained/oriented how to use rectal Artesunate?**		□ Yes	□ No
If yes, date of training	**day|____|____| month|____|____| year |____|____|**		
**Have you ever been trained/oriented on prereferral management of severe malaria?**		□ Yes	□ No
	**day|____|____| month|____|____| year |____|____|**		
**Have you ever been trained/oriented on Emergency Triage, Assessment and Treatment?**		□ Yes	□ No
If yes, date of training	**day|____|____| month|____|____| year |____|____|**		

**GUIDELINES**		
Do you have access to **any malaria guideline** for HWs? (Y/N)	□ Yes	□ No
If yes, which edition		
Do you have access to **Uganda Clinical Guidelines**	□ Yes	□ No
If yes, which edition		
Do you have access to **IMCI booklet**	□ Yes	□ No
If yes, which edition		

**SUPERVISION**			
**Did you have** any supervisory visit **in the** last 3 months		□ Yes	□ No
If yes, was malaria case management topic of **any of these visits?**		□ Yes	□ No
If yes, was any of these activities done?	Review of malaria records and registers?	□ Yes	□ No
	Discussion with supervisor on malaria case management?	□ Yes	□ No
	Support about artesunate use?	□ Yes	□ No
	Observation of outpatient consultations?	□ Yes	□ No
	Provision of feedback?	□ Yes	□ No
	Other, specify	□ Yes	□ No
	Other, specify	□ Yes	□ No

**KNOWLEDGE ABOUT MALARIA CASE MANAGEMENT POLICIES (self-administered)**				
All patients with fever or history of fever should be tested for malaria		□ True	□ False	□ Don't Know
Only patients who test positive should be treated for malaria		□ True	□ False	□ Don't Know
Would you classify this area as high, low or Medium malaria risk area	□ High	□ Low	□ Medium	□ Don't know

What is the **1st line treatment** for uncomplicated malaria for **children above 5kg and adults**		□ Don't know
What is the **1st line treatment** for uncomplicated malaria for **children below 5kg**		□ Don't know
What is the alternative 1st line treatment for uncomplicated malaria		□ Don't know
What is the 2nd line treatment for uncomplicated malaria		□ Don't know
What is the 1st line treatment for uncomplicated malaria for **pregnant women in the 1st trimester**		□ Don't know
What is the 1st line treatment for uncomplicated malaria for **pregnant women in 2nd/ 3rd trimester**		□ Don't know
What is the 1st line treatment recommended for treatment of **severe malaria**?		□ Don't know
What is the drug recommended **for continuing treatment** of severe malaria after the initial phase?		□ Don't know
What is the 2nd line treatment for severe malaria		□ Don't know

KNOWLEDGE ABOUT PRE-REFERRAL MALARIA MANAGEMENT (self-administered)	
What is the recommended drug for **pre-referral treatment** of suspected **severe malaria** for **children and non-pregnant adults**?	
	□ Don't know
What is the name of recommended drug for pre-referral management of suspected severe malaria for **pregnant women**?	
	□ Don't know
What antimalarial drug do **you usually give** before referring **child** with suspected or confirmed severe malaria?	
	□ Don't know
What antimalarial drug do **you usually give** before **referring child** with suspected or confirmed severe malaria?	
□ I have never referred child with suspected or confirmed severe malaria	
□ I refer patient with letter/notes but I usually do not give antimalarial drugs before referral	
□ I give antimalarials, specify antimalarial given………………………………………………	
□ I give antimalarials, but don't know the name	

What is the **preferred route of artesunate administration** for pre-referral management of suspected severe malaria?				
□ Oral	□ Intravenous (IV)	□ Intramuscular (IM)	□ Rectal (PR)	□ Other, specify

What solution should be first used to **reconstitute artesunate powder**?			
□ Water for injection	□ Ringers (Hartman's)	□ Bicarbonate (5% sodium bicarbonate)	□ Don't know
□ Normal saline (sodium chloride)	□ 5% Dextrose	□ Half strength Darrows (HSD)	□ Other,…………………..

What is the recommended dose of Artesunate for a child **less than 20kg**? [Circle **only one** response]			
□ 2.0 mg/kg	□ 2.4 mg/kg	□ 3.0 mg/kg	□ Don't know
□ 3.6 mg/kg	□ 4.2 mg/kg	□ 5.0 mg/kg	□ Other dose,………………..

What is the recommended dose of Artesunate for a child **over 20kg**? [Circle **only one** response]			
□ 2.0 mg/kg	□ 2.4 mg/kg	□ 3.0 mg/kg	□ Don't know
□ 3.6 mg/kg	□ 4.2 mg/kg	□ 5.0 mg/kg	□ Other dose,……………………..

What are the clinical features indicating **severe malaria** requiring outpatient referral of the patient? (check all mentioned)	
□ Severe pallor	□ Respiratory distress
□ Severe anemia	□ Abdominal pain
□ Convulsions	□ Prostration (Extreme weakness, unable to sit or stand without support)
□ Renal failure	□ Coma
□ Moderate anemia	□ Joint pains
□ Vomiting everything	□ Severe abdominal pain
□ Unconsciousness	□ Failure to breast feed
□ Headache	□ Jaundice

Do you usually refer patients with severe malaria (suspected or confirmed)							□ Yes	□ No
If yes,	specify the name and level of health facility							
	specify the level of health facility	□ National Referral	□ Regional referral	□ District Hospital	□ HCIV	□ HCIII	□ HCII	

**Knowledge about Artesunate preparation and administration**		
Have you ever prepared and administered IV Artesunate before referral of the patient?	□ Yes	□ No
Do you know how to prepare and administer IV Artesunate?	□ Yes	□ No
Have you ever administered Artesunate suppository intrarectally before referral of patient	□ Yes	□ No

Do you know how to administer Artesunate suppository intrarectally?	□ Yes	□ No
Have you ever prepared and administered IM Artesunate before referral of the patient? (Y/N)	□ Yes	□ No
Do you know how to prepare and administer IM Artesunate before referral of the patient?	□ Yes	□ No

CASE SCENARIO	
**If you know how to prepare and administer IV ARTESUNATE answer the questions below**	
**Case scenario 1: IV Artesunate 60mg/vial for Child 9 kg**	
How many **vials of artesunate** do you need per dose? [number]	
How many **mls of bicarbonate** do you need to reconstitute artesunate? [number]	
How many **mls of normal saline or 5% dextrose** you need to add to reconstituted artesunate? [number]	
How many **mls of prepared artesunate** solution you should administer? [number]	
What is the minimum number of doses of IV ARTESUNATE you would give the child	
**Case scenario 2: IV Artesunate 60mg/vial for an Adult 80 kg**	
How many **vials of artesunate** do you need per dose? [number]	
How many **mls of bicarbonate** do you need to reconstitute artesunate? [number]	
How many **mls of normal saline or 5% dextrose** you need to add to reconstituted artesunate? [number]	
How many **mls of prepared artesunate** solution you should administer? [number]	
What is the minimum number of doses of IV ARTESUNATE you would give the child	

COVID-19 QUESTIONS (KNOWLEDGE ASSESSMENT)			
**Have you had any COVID-19 specific training**		□ Yes	□ No
If yes**,**	**day|_____|_____| mo|_____|_____| year |_____|_____|**		
**If yes, what topics were covered (tick all that apply)**	□ Etiology (the cause of COVID)	□ Symptoms and Signs	□ Screening<br>□ High risk groups
	□ Transmission (How COVID-19 is spread)	□ Case definition	□ How to use a mask
	□ Treatment of cases	□ Others, specify	
**If yes name of training organization**			

1	COVID-19 is caused by<br>(one answer is correct)	A	By a new virus (Severe Acute Respiratory Syndrome coronavirus 2
		B	By a bacterium
		C	By a fungus
		D	By an arbovirus (Yellow fever virus)
2	COVID-19 is spread by<br>(one answer is correct)	A	Touching surfaces contaminated by respiratory droplets of an infected person
		B	Drinking dirty water
		C	Mosquitoes
		D	Skin to skin contact
3	A COVID-19 suspect must be<br>(only one answer is correct)	A	Sent to the laboratory for urinalysis
		B	Isolated with immediate effect
		C	Triaged as non-urgent and waits his turn to be seen by the healthcare worker
		D	Given paracetamol as they wait to be seen

**FOR EACH QUESTION TICK ALL THAT APPLY**				
4	A patient with COVID-19 presents with any of the following symptoms (Tick all that apply)	□ Fever	□ Diarrhea	□ Shortness of breath
		□ Flu	□ Muscle pain	□ Sore throat
		□ Cough	□ Nausea	□ Loss of smell
		□ Headache	□ Renal failure	□ Disseminated intravascular coagulation
5	The following are methods of preventing spread of COVID-19 at a health facility (Tick all that apply)	□ Hand washing with soap		□ Avoid touching eyes, mouth and nose
		□ Hand washing with alcohol (70%)		□ Sneezing into elbow
		□ Cleaning surfaces with chlorine solution		□ Avoid crowding
		□ Observing social distance		□ Isolating suspects
		□ Wearing cloth or medical masks		□ Screening patients for fever

**FOR EACH STATEMENT PROVIDE ANSWER: TRUE OR FALSE OR I DON'T KNOW**				
6	COVID-19 is a highly infectious disease	□ True	□ False	□ I don't know
7	Children are not affected by COVID-19	□ True	□ False	□ I don't know
8	Older people are more likely to die of COVID-19 than younger people	□ True	□ False	□ I don't know
9	COVID-19 can be spread by drinking un-boiled water	□ True	□ False	□ I don't know
10	People with Diabetes Mellitus are at increasing risk of dying of COVID-19	□ True	□ False	□ I don't know
11	It's not necessary for children to take measure to prevent infection by COVID-19	□ True	□ False	□ I don't know
12	Only using medical mass will protect me from getting COVID-19	□ True	□ False	□ I don't know
13	Non reusable medical masks can be worn for more than 1 days	□ True	□ False	□ I don't know
14	Wearing face shields of goggles are an effective in prevention of eye contamination	□ True	□ False	□ I don't know
15	Contacts of confirmed cases of COVID-19 should be isolated	□ True	□ False	□ I don't know
16	COVID-19 can be cured by herbs	□ True	□ False	□ I don't know
17	COVID-19 can be cured by common medicines (Chloroquine and Vitamin C)	□ True	□ False	□ I don't know
18	If anyone get the novel coronavirus (SAR-COV-19), there is no possibility of survival.	□ True	□ False	□ I don't know
19	You only need to wash your hands when they are visibly dirty	□ True	□ False	□ I don't know

**COVID-19 PRACTICES ATTITUDES AND FEARS**						
While on duty do you wear a mask					□ Yes	□ No
If yes,	**Type of mask**	□Cloth mask	□ N95	□ Non-reusable medical mask	□ Rarely	
	**How often**	□ All the time	□Some of the time	□ Other, specify		
What protective equipment do you use while on duty at the facility **(tick all that apply)**	□ Gloves	□ Gown	□ Heavy duty gloves	□ N95	□ Medical masks	□ Cloth mask
	□ Goggles	□ Apron	□ Alcohol (70%) hand rub	□ JIK (Chlorine)	□ Face shield	□ Boots
While on duty do you have access to hand washing facilities					□ Yes	□ No
If not, state the reason						
While on duty do you feel **at risk** of contracting COVID-19					□ Yes	□ No
If not, state the reason						
Are you **afraid** of contracting COVID-19					□ Yes	□ No
If yes, state the reasons						
If no, state the reasons						
