# Supplementary material for: Adherence to malaria management guidelines by health care workers in the Busoga sub-region, eastern Uganda
Source: Malar J. 2022 Jan 25;21:25. doi: 10.1186/s12936-022-04048-2 (PMC8788114; doi:10.1186/s12936-022-04048-2)
Supplement: Supplementary file 3 — Additional file 3: The patient exit interview questionnaire. The questionnaire was transformed into an electonic version enabling collection of data using tablets. [file 12936_2022_4048_MOESM3_ESM.pdf]

Additional file 3: Patient exit interview questionnaire

<table>
<tr><td>Name of facility</td><td colspan="8"></td></tr>
<tr><td>Level</td><td>□ NRH</td><td>□ RRH</td><td>□ RH</td><td>□ GH</td><td>□ HCIV</td><td>□ HCIII</td><td>□ HCII</td><td>□ HCI</td></tr>
<tr><td>Ownership</td><td colspan="2">□ GOU</td><td colspan="2">□PNFP</td><td colspan="2">□ PFP</td><td colspan="2">□ Community</td></tr>
<tr><td>Patient #</td><td colspan="2">|____|____|____|</td><td>Facility code #</td><td colspan="2">|____|____|____|</td><td>District code #</td><td colspan="2">|____|____|____|</td></tr>
<tr><td>Patient initials</td><td></td><td>Patient age *</td><td colspan="2"></td><td>Patient sex</td><td>□ Male</td><td colspan="2">□ Female</td></tr>
<tr><td>Date of completion</td><td colspan="8">day|_____|_____| month|_____|_____| year |_____|_____|</td></tr>
<tr><td>Unique ID</td><td colspan="8">Patient number #: |____|____|____| Facility Code: |____|____|____| Must be same as above District Code |____|____|____| Must be same as above</td></tr>
<tr><td>Name of data collector</td><td colspan="8"></td></tr>
</table>

RAPID SCREENING		
Was patient **referred** to another facility for hospitalisation?	□ Yes	□ No
Was patient **admitted** to this facility for hospitalisation?	□ Yes	□ No
Is this patient's **follow up visit** for the same illness?	□ Yes	□ No
Is patient's **weight less than 5 kg**?	□ Yes	□ No
Is patient presenting **without fever** during this illness?	□ Yes	□ No
Is patient **likely to be pregnant**?	□ Yes	□ No
Have any of the boxes shaded grey been ticked, if yes exclude from the study	□ Yes	□ No

HISTORY AND MEASUREMENTS		
~~Patients initials~~		
~~Patients age (cross check with date of birth on immunization card, where applicable)~~		
~~Patient gender~~	□ Male	□ Female
Patients temperature		
Does the patient present illness involve a fever	□ Yes	□ No
If a child, does present illness involve any danger sign	□ Yes	□ No
If yes, specify: □ inability to drink or breastfeed, □ vomiting everything □ history of convulsions during the current illness □ convulsions now □ lethargy or unconsciousness □ Other, specify		
Was fever present in last 48 hours?	□ Yes	□ No
How many illness episodes with fever in past 1 month?		
Duration of current illness in days		

PATIENT MAIN COMPLAINTS (LIST ALL THAT APPLY)	
1.	6.
2.	7.
3.	8.
4.	9.
5.	10.
11.	12.

PRIOR USE OF ANTIMALARIALS				
Did patient take any **antimalarial** for this illness **PRIOR** to this visit (limited to current illness)?			□ **Yes**	□ **No**
If, yes	Name formulation (check if patient has some)			
	When was the first dose taken (count backwards/retrospectively, today being day 1)			
	When was the last dose taken (count backwards/retrospectively, today being day 1)			
	Number of doses (scheduled treatment) taken in total			
	Number of tablets taken in total			
	Was it a complete dose		□ Yes	□ No
	Where was the antimalarial obtained from	□ HCII	□ HCIII	□ HCIV	□ Hospital
		□ Self-medication	□ Drug shop/Pharmacy	□ Private Clinic
		□ Other, specify,		
Did patient take a second **antimalarial** for this illness **PRIOR** to this visit?			□ Yes	□ No
	Name formulation (check if patient has some)			
	When was the first dose taken (count backwards/retrospectively, today being day 1			
	When was the last dose taken (count backwards/retrospectively, today being day 1			
	Number of doses taken in total			
	Number of tablets taken in total			
	Was it a complete dose		□ Yes	□ No
	Where was the antimalarial obtained from	□ HCII	□ HCIII	□ HCIV	□ Hospital
		□ Self-medication	□ Drug shop/Pharmacy	□ Private Clinic
		□ Other, specify,		
**ROUTINE HEALTH WORKER PRACTISE**				
Did any health worker **ask/record patients age** during this visit			□ Yes	□ No
Did any health worker **measure the weight?**			□ Yes	□ No
Did any health worker **measure temperature?**			□ Yes	□ No
Did any health worker ask **about previous use of antimalarials?**			□ Yes	□ No
**LABORATORY**				
Was the patient sent for **malaria blood slide (microscopy)**			□ Yes	□ No
If yes as a **malaria blood slide (microscopy**) test done			□ Yes	□ No
If yes, what was the result			□ Positive	□ Negative
Was a malaria RDT done			□ Yes	□ No
If yes, what was the result			□ Positive	□ Negative
**Laboratory report (write all other tests done, and results. If none write NONE**				

**DIAGNOSIS AND TREATMENT**			
**Patients diagnosis (write all diagnosis as is written in the patients' cards If none write NONE**			
**Treatment (write all treatments and prescriptions as is. If none write NONE**			
**ANTIMALARIAL DRUG PRESCRIBED/DISPENSED**			
Was the patient prescribed antimalarials (ASK TO SEE MEDICINE)		□ Yes	□ No
If, yes	Name formulation of medicine		
	Was the medicine dispensed (given) to the patient at the facility	□ Yes	□ No
	Was the first dose administered at the facility	□ Yes	□ No
	Was the first dose swallowed in front of the health worker	□ Yes	□ No
	Did any of the health workers explain to you how to take the medicine at home	□ Yes	□ No
	Did any of the health workers tell you to take/give the next does after 8 hours	□ Yes	□ No
	Did any of the health workers tell you to take/give medicine after meal or food	□ Yes	□ No
	Were you told to complete the medicine even if you/your child feels better	□ Yes	□ No
	Were you advised what to do in case of a drug reaction	□ Yes	□ No
	Were you advised what to do if the child vomited	□ Yes	□ No
If yes, what were you advised			
Was more than one antimalarial prescribed to the patient		**□ Yes**	**□ No**
If, yes	Name formulation		
	When was the first dose taken (count backwards/retrospectively, today being day 1		
	Was the first dose administered at the facility	□ Yes	□ No
	Was the first dose swallowed in front of the health worker	□ Yes	□ No
	Did any of the health workers explain to you how to take the medicine at home	□ Yes	□ No
	Did any of the health workers tell you to take/give the next does after 8 hours	□ Yes	□ No
	Did any of the health workers tell you to take/give medicine after meal or food	□ Yes	□ No
	Were you told to complete the medicine even if you/your child feels better	□ Yes	□ No
	Were you advised what to do in case of a drug reaction	□ Yes	□ No
	Were you advised what to do if the child vomited	□ Yes	□ No
If yes, what were you advised			

**DISPENSED AL (complete this section if patient was given AL at the facility)**			
Was the patient given ORIGINAL, not cut AL pack (observe)		◻ Yes	◻ No
If yes, specify which blister pack	**Type of pack**	**Brand name**	**Number of blisters**
	◻ AL 6 pack		
	◻ Al 12 pack		
	◻ AL 18 pack		
	◻ AL 24 pack		
Was the patient given any **CUT AL** blister pack?		◻ Yes	◻ No
If yes, describe which pack was cut and how AL dose was dispensed			
Was the patient given any **loose AL tablets**?		◻ Yes	◻ No
Was the patient dispensed/given **dispersible AL**		◻ Yes	◻ No
If yes, was **AL administered dispersed in the water**		◻ Yes	◻ No
If yes, was mother instructed on how to give AL dispersed in water		◻ Yes	◻ No

**CUSTOMER CARE/CLIENT SATISFACTION WITH SERVICE**					
What time did you arrive at the facility				|____|____|:|____|____|	
Upon arrival, was the outpatient department open				◻ Yes	◻ No
Did a health worker greet you?				◻ Yes	◻ No
If yes, at what point					
Did any of HWs speak to you rudely/shout at you				◻ Yes	◻ No
What time were **first seen and evaluated** by a health care worker for your illness				|____|____|:|____|____|	
Were you sent to the laboratory	◻ Yes	◻ No	If yes, were you offered a service at the lab	◻ Yes	◻ No
If yes,	What time did you arrive at the lab			|____|____|:|____|____|	
	What time were you attended to at the lab			|____|____|:|____|____|	
	What time were you results returned			|____|____|:|____|____|	
Were you sent to the pharmacy	◻ Yes	◻ No	If yes, were you given medicine at the pharmacy	◻ Yes	◻ No
If yes,	What time did you arrive at the pharmacy			|____|____|:|____|____|	
	What time did you receive your medicine			|____|____|:|____|____|	
Were you given all the prescribed medicine				◻ Yes	◻ No
Were you charged for any service at the facility	◻ Yes	◻ No	If yes, overall, how much did you pay		
What time did you leave the facility				|____|____|:|____|____|	
Rate satisfaction with services offered on a scale of 0(worst) to 10(best)				|____|____| **out of 10**	

**REPEAT ANTIMALARIAL TEST**					
Was a malaria RDT done	◻ Yes	◻ No	If yes, what was the result	◻ Positive	◻ Negative
Was a blood smear (microscopy) done	◻ Yes	◻ No	If yes, what was the result	◻ Positive	◻ Negative
Was a filter paper sample collected				◻ Yes	◻ No
