# Supplementary material for: Adherence to malaria management guidelines by health care workers in the Busoga sub-region, eastern Uganda
Source: Malar J. 2022 Jan 25;21:25. doi: 10.1186/s12936-022-04048-2 (PMC8788114; doi:10.1186/s12936-022-04048-2)
Supplement: Supplementary file 8 — Additional file 8: A table showing HCW interview response rates stratified by staff position and grouped into HCWs targeted (directly involved in providing patient care) and those not targeted (indirectly involved in providing patient care) for interviews. [file 12936_2022_4048_MOESM8_ESM.docx]

Additional file 8: Health care worker response rates for interviews

| **Position** | **Participated in HCW interviews**  **(a)** | **Staff on site on the day of the assessment**  **(b)** | **Response  rate (%)**  **(a/b X 100)** |
| --- | --- | --- | --- |
| **Directly involved in patient care** | | | |
| Doctor | 11 | 37 | 30% |
| Clinical Officer | 154 | 230 | 67% |
| Registered Nurse | 177 | 219 | 81% |
| Enrolled Nurse | 507 | 747 | 68% |
| Nurse assistant | 178 | 241 | 74% |
| **Sub total** | **1027** | **1474** | **70%** |
| **Not directly involved in patient care** | | | |
| Laboratory technology | 32 | 272 | 12% |
| Other | 27 | 638 | 4% |
| **Sub total** | **59** | **910** | **6%** |
| **TOTAL** | **1086** | **2384** | **46%** |

HCW; Health care worker
