# Supplementary material for: Adherence to malaria management guidelines by health care workers in the Busoga sub-region, eastern Uganda
Source: Malar J. 2022 Jan 25;21:25. doi: 10.1186/s12936-022-04048-2 (PMC8788114; doi:10.1186/s12936-022-04048-2)
Supplement: Supplementary file 9 — Additional file 9: Generalized estimating equation (GEE) model was used to identify independent patient, facility, and health care worker factors associated with appropriate malaria case management defined in three different ways based on varying treatment options for confirmed malaria: (a) AL prescribed, (b) ACT prescribed, or (c) AL given to the patient. [file 12936_2022_4048_MOESM9_ESM.docx]

Additional file 9. Adjusted analysis of factors associated with appropriate malaria case management

| **Variable** | **Different definitions of appropriate malaria case management** | | | | | |
| --- | --- | --- | --- | --- | --- | --- |
|  | **Appropriate malaria case management**  **(AL prescribed)** | | **Appropriate malaria case management**  **(ACT prescribed)** | | **Appropriate malaria case management**  **(AL given)** | |
|  | **OR (95% CI)** | **p-value** | **OR (95% CI)** | **p-value** | **OR (95% CI)** | **p-value** |
| **Patient characteristics** |  |  |  |  |  |  |
| Age in years | 1.01 (1.01, 1.02) | 0.002 | 1.01 (1.01, 1.02) | <0.001 | 1.01 (1.01, 1.02) | 0.002 |
| Prior use of antimalarials |  |  | 0.84 (0.62, 1.15) | 0.05 |  |  |
| **Documentation of information on patient record** |  |  |  |  |  |  |
| Age and prior use of antimalarials |  |  |  |  | 1.07 (0.87, 1.33) | 0.483 |
| Temperature and weight | 2.07 (1.46, 2.93) | <0.001 | 2.46 (1.73, 3.50) | <0.001 | 2.00 (1.57, 2.55) | <0.001 |
| Patient satisfaction score | 1.12 (1.06, 1.18) | <0.001 | 1.12 (1.07, 1.18) | < 0.001 | 1.10 (1.05, 1.14) | <0.001 |
| **Health facility characteristics** |  |  |  |  |  |  |
| **Level** |  |  |  |  |  |  |
| Health centre IV | 1 |  | 1 |  | 1 |  |
| Health centre III | 6.86 (1.57, 29.8) | 0.010 | 6.34 (1.11, 36.0) | 0.340 | 3.03 (0.55, 16.5) | 0.200 |
| Health centre II | 8.29 (2.36, 29.0) | 0.001 | 6.74 (1.83, 24.7) | 0.735 | 4.55 (0.98, 21.0) | 0.052 |
| **Owner** |  |  |  |  |  |  |
| GOU | 1 |  | 1 |  | 1 |  |
| PNFP | 0.55 (0.32, 0.97) | 0.041 | 0.47 (0.26, 0.86) | 0.015 | 0.67 (0.40, 1.15) | 0.041 |
| PFP | 0.21 (0.10, 0.42) | < 0.001 | 0.26 (0.12, 0.58) | 0.001 | 0.23 (0.13, 0.42) | < 0.001 |
| Supervised in MCM 2019/2020 | 4.39 (1.62, 11.9) | 0.003 | 4.00 (1.43, 11.1) | < 0.001 | 4.54 (2.08, 9.90) | < 0.001 |
| **Availability of equipment / medicines** |  |  |  |  |  |  |
| AL in stock on survey day | 2.27 (0.52, 9.85) | 0.271 | 2.05 (0.46, 9.04) | 0.341 | 2.72 (0.48, 15.3) | 0.255 |
| Malaria test on survey day | 7.73 (3.84, 15.6) | < 0.001 | 6.55 (2.91, 14.7) | 0.001 | 2.99 (1.45, 6.18) | 0.003 |
| **Availability of guidelines/charts** |  |  |  |  |  |  |
| IMCI guidelines | 0.88 (0.51, 1.52) | 0.670 | 0.93 (0.51, 1.68) | 0.827 | 0.47 (0.28, 0.77) | 0.003 |
| UCG guidelines | 3.28 (1.96, 5.49) | < 0.001 | 3.21 (1.82, 5.68) | < 0.001 | 2.42 (1.51, 3.86) | < 0.001 |
| **Health care worker characteristics** |  |  |  |  |  |  |
| Supervised in MCM in the past 3 months | 1.64 (1.18, 2.28) | 0.003 | 1.65 (1.14, 2.38) | 0.007 | 1.77 (1.34, 2.32) | < 0.001 |
| Access to UCG | 1.25 (0.90, 1.72) | 0.174 | 1.15 (0.82, 1.61) | 0.409 | 1.07 (0.83, 1.38) | 0.575 |
